# Response of *Sorghum bicolor* genotypes for yield and yield components and organic carbon storage in the shoot and root systems

**DOI:** 10.1038/s41598-024-59956-x

**Published:** 2024-04-25

**Authors:** Asande Ngidi, Hussein Shimelis, Seltene Abady, Sandiswa Figlan, Vincent Chaplot

**Affiliations:** 1 African Centre for Crop Improvement, School of Agricultural, Earth and Environmental Sciences, University of KwaZulu-Natal, Private Bag X01, Scottsville, Pietermaritzburg, 3209 South Africa; 2 Department of Agriculture and Animal Health, University of South Africa, Florida, 1709 South Africa; 3 School of Agricultural, Earth and Environmental Sciences, University of KwaZulu-Natal, Private Bag X01, Scottsville, Pietermaritzburg, 3209 South Africa; 4 Laboratory of Oceanography and Climate, Experiments and Numerical Approaches (LOCEAN), UMR 7159, IRD/C NRS/UPMC/MNHN, IPSL, 75005 Paris, France

**Keywords:** Biological techniques, Plant sciences

## Abstract

Sorghum is a vital food and feed crop in the world’s dry regions. Developing sorghum cultivars with high biomass production and carbon sequestration can contribute to soil health and crop productivity. The objective of this study was to assess agronomic performance, biomass production and carbon accumulation in selected sorghum genotypes for production and breeding. Fifty sorghum genotypes were evaluated at three locations (Silverton, Ukulinga, and Bethlehem) in South Africa during 2022 and 2023 growing seasons. Significant genotype × location (*p* < 0.05) interactions were detected for days to 50% heading (DTH), days to 50% maturity (DTM), plant height (PH), total plant biomass (PB), shoot biomass (SB), root biomass (RB), root-to-shoot biomass ratio (RS), and grain yield (GY). The highest GY was recorded for genotypes AS115 (25.08 g plant^−1^), AS251 (21.83 g plant^−1^), and AS134 (21.42 g plant^−1^). Genotypes AS122 and AS27 ranked first and second, respectively, for all the carbon stock parameters except for root carbon stock (RCs), whereas genotype AS108 had the highest RCs of 8.87 g plant^−1^. The principal component analysis identified GY, DTH, PH, PB, SB, RB, RCs, RCs/SCs, total plant carbon stock (PCs), shoot carbon stock (SCs), and grain carbon stock (GCs) as the most discriminated traits among the test genotypes. The cluster analysis using agronomic and carbon-related parameters delineated the test genotypes into three genetic groups, indicating marked genetic diversity for cultivar development and enhanced C storage and sustainable sorghum production. The selected sorghum genotypes are recommended for further breeding and variety release adapted to various agroecologies in South Africa.

## Introduction

Sorghum (*Sorghum bicolar* [L.] Moench, 2n = 2x = 20) is the fifth most important cereal crop after maize, wheat, rice, and barley cultivated globally. It is the primary food source for approximately 750 million people living in the semi-arid tropics of Africa, Asia, and Latin America, with an annual global production of 61.62 million tonnes^1^. It serves as a raw material for feed, bioenergy, and industrial applications. It has unique nutritional value and adaptation to dryland conditions. In South Africa, sorghum is one of the most widely grown crops with an annual production of 215,000 tons^2^. In the country, the largest sorghum production is found in Mpumalanga (41%) followed by Limpopo (34%), Free State (31%), and North West (25%) Provinces. In sub-Saharan Africa, sorghum is predominantly produced by smallholder farmers who have limited access to production inputs such as improved seeds, crop protection chemicals, inorganic fertilizers, irrigation facilities, and finance^3^. Furthermore, the major proportion of sorghum is cultivated under marginal and poor soil conditions, resulting in low crop yields and quality.

Reportedly, sorghum is one of the major crops with high biomass production with a substantial capacity for carbon (C) storage in agricultural soils^4^. Improved sorghum cultivars with high biomass production and carbon sequestration can contribute to soil health and crop productivity. Agricultural soils cover up to 34% of the global land surface^5^. Hence, adequate management of agricultural lands is vital to maintaining soil organic C storage by incorporating crop biomasses and residues with enhanced decomposition^6,7^.

There is a high carbon loss in cultivated croplands in Africa due to the removal of most plant residues after harvest for various household uses (e.g. fuel wood, livestock feed, and construction material). Also, high biomass decomposition rates and nutrient loss due to erosion causes poor soil fertility and low yield gains^8^. Approximately 17% of all atmospheric CO_2_ flows through the plant-soil-atmosphere interaction each year, making soil and plant C critical to the global C cycle^9^. Root C is a major contributor to soil organic C, accounting for up to 90% of all C inputs to arable soils^10^. Due to its unique and stable chemical composition^11^ and partitioning into more stable components^12^, root C has more extended residence in the soil bank compared to C-derived from above-ground crop residue^10,13,14^. Plant roots have relatively low decomposer association and high C storage capacity in deep soil layers^15,16^, serving as a long-term C reservoir^17,18^. Therefore, crop ideotypes such as sorghum genotypes with fibrous and deep root systems have been identified as promising contributors to enhanced carbon in soils, with an estimated potential to sequester 1 Pg yr^−1^ of atmospheric CO_2_^7,19,20^.

There is a marked difference among crops and genotypes within a crop in C storage and deposition of plant C into soils. For instance, Mathew^21^ reported that barley transferred 29% C into the soil, followed by maize (20%), and wheat (18%). Similarly, Bolinder^22^ reported higher (50%) annual C transferred to the soil by barley compared to oats (48%) and winter wheat (32%). Manna^23^ also reported a 45% C transfer into the soil by sorghum as compared to 33% by rice under different soil types. Significant differences for C storage by wheat cultivars have also been reported^24^, where the authors found that plant C stocks under 25% field capacity were the highest for genotypes BW152 (1059 g C m^−2^) and BW141 (1004 g C m^−2^), while genotype BW140 and LM26 had values below 850 g C m^−2^, and genotype BW141 had the highest plant C stock of 2260 g C m^-2^ under 75% field capacity. Ahmed^25^ reported that Wary sorghum genotype exhibited the highest root C allocation at 386 Mg C ha^-1^, while UNL-hybrid-5 demonstrated the lowest root C allocation at 140 Mg C ha^-1^. Moreover, the authors reported that genotype BATAEM-4 exhibited the highest shoot C allocation at 3334 Mg C ha^-1^, whereas UNL-hybrid-5 exhibited the lowest shoot C allocation, measured at 1007 Mg C ha^-1^ at different locations. Xiang^4^ reported that growing sorghum varieties with high biomass could significantly increase C sequestration in soils. In another study by Liang^26^, maize varieties had high yield potential and greater capacity to store C in plant biomass and soil. Amujoyegbe^27^ reported increased root biomass and root C stocks allocation in maize by 35% and sorghum by 18.2% when the soil nitrogen content increased. From the above literature, it is necessary to evaluate specific crop types and genotypes in the target production environments for the targeted recommendation and devise the best management practices that can be adopted to increase biomass allocation to the roots for land rehabilitation, soil C storage and crop productivity.

Mangena^28^ reported significant differences for agronomic traits and biomass production between sorghum genotypes evaluated, where the use of 190 diverse sweet sorghum genotypes played a crucial role in shaping the observed variations. The authors found that biomass yield varied from 6.67 to 111.20 t ha^-1^, with genotype AS203 producing 20% higher yield than all other genotypes. Abraha^29^ reported that under drought conditions, sweet sorghum genotypes EG 469 and Hamelmalo had the highest biomass production of 16.70 t ha^-1^ and 18.10 t ha^-1^ and produced the highest grain yields of 2.70 t ha^-1^ and 2.6 t ha^-1^, respectively. Complementary and contrasting genotypes can be used to create new breeding populations to develop a sorghum ideotype characterized by high root biomass and improved yield gains.

There is limited knowledge that documented the genetic diversity of sorghum integrating agronomic traits, balanced biomass allocation, and C sequestration under South African production conditions. Previous studies have reported differences in plant C stocks between crop types with limited emphasis on intra-specific variations to guide breeding, especially for C sequestration potential. Screening genetically diverse sorghum lines will enable the selection of best-performing genotypes for crop breeding and selection programs. Sorghum genotypes with desirable agronomic traits and high biomass production can improve C sequestration capability and yield gains through enhanced water and nutrient use efficiencies. Therefore, the objective of this study was to assess agronomic performance, biomass production and carbon accumulation in genetically diverse sorghum lines across three different locations to select unique genotypes for production and breeding. The findings may be beneficial for crop breeders to assess the variations in biomass allocation and agronomic performances. This is crucial for developing sorghum genotypes with increased grain yield, drought tolerance, water use efficiency, and the capacity for C sequestration into soils.

## Results

### Analysis of variance for agronomic traits

Combined analysis of variance revealed significant (*p* < 0.05) differences among the sorghum genotypes for all the assessed agronomic traits except DTM (Table 1). Significant genotype by location (*p* < 0.05) interactions were recorded for DTH, DTM, PH, PB, SB, RB, RS, and GY.

**Table 1:** Combined analysis of variance and significance tests for agronomic traits of 50 sorghum genotypes across three locations in South Africa.

Souce of variation	DF	DTH	DTM	PH	PB	SB	RB	RS	GY	HI
Location	2	91.83	349.41	20,546.17***	20.14	18.49	22.83**	0.004	13.70**	50.34
Replication	3	479.85	519.62**	28,833.24***	9.61	7.93	0.37	0.01	7.43*	18.37
Block	24	167.77	111.44	2757.55	25.17	11.53	3.31	0.005	4.42*	21.91
Genotype	49	307.42*	130.78	3194.9**	349.57***	151.72***	129.22***	0.26***	181.77***	1031.47***
Genotype × Location	98	207.06**	111.63*	2257.43**	18.36***	14.44**	4.5*	0.004*	2.57***	15.34
Error	123	195.51	135.71	1826.3	15.99	14.52	5.06	0.004	2.73	17.83
CV (%)		17.58	8.44	27.39	13.25	23.36	9.35	12.46	13.88	15.53
LSD (5%)		15.97	13.22	48.55	5.09	4.24	2.4	0.28	1.98	5.94

*, ** and *** denote significant at *p* < 0.05, *p* < 0.01, and *p* < 0.001, respectively. SOV = source of variation, DF = degrees of freedom, CV = coefficient of variation, LSD = least square significance at 5%, DTH = days to 50% heading, DTM = Days to 50% maturity, PH = plant height (cm), PB = total plant biomass (g plant^-1^) , SB = shoot biomass (g plant^-1^), RB = root biomass (g plant^-1^), RS = root to shoot biomass ratio, GY = grain yield (g plant^-1^), HI = harvest index (%).

### Analysis of variance for carbon storage

The analysis of variance test genotypes for carbon parameters revealed significant differences (*p* < 0.001) among test genotypes for GCc, PCs, SCs, RCs, RCs/SCs, and GCs (Table 2).

**Table 2:** Analysis of variance and significance tests for carbon storage of the 25 selected sorghum genotypes at Silverton during 2022 growing season.

Source of variation	DF	SCc	RCc	GCc	PCs	SCs	RCs	RCs/SCs	GCs
Replication	1	1.24*	5.21	0.03	1.34	2.85	0.29	0.004	0.86
Block	8	0.28**	7.45**	0.01**	10.88	13.41	0.38	0.05*	0.21
Genotype	24	0.65	12.77	0.49***	29.02**	20.13*	5.47***	0.13***	8.44**
Error	16	0.32	8.03	0.01	8.76	7.61	0.42	0.02	0.34
CV		1.28	6.9	0.28	23.14	33.64	14.05	20.72	11.65
LSD (5%)		1.11	5.13	0.23	6.28	6.04	1.34	0.29	0.91

*, ** and *** denote significant at *p* < 0.05, *p* < 0.01, and *p* < 0.001, respectively. SOV = source of variation, DF = degrees of freedom, CV = coefficient of variation, LSD = least square significance at 5%, SCc = shoot carbon content (%), RCc = root carbon content (%), GCc = grain carbon content (%), PCs = total plant carbon stocks (g plant^-1^), SCs = shoot carbon stock (g plant^-1^), RCs = root carbon stock (g plant^-1^), RCs/SCs = root to shoot carbon stock ratio, GCs = grain carbon stock (g plant^-1^).

### Performance of sorghum genotypes for agronomic traits and carbon allocation

#### Agronomic performance

The mean performance of sorghum genotypes for nine agronomic traits across three locations is summarized in Table 3. The mean DTH and DTM were 80 and 138 days, respectively. Genotype G50 was the earliest to reach 50% heading and maturity at 61 and 114 days, respectively, followed by AS72 (69 and 133 days), AS122 (69 and 139 days), AS141 (69 and 137 days), and AS117 (70 and 139 days) (Table S1). Extended flowering and maturity periods were recorded for genotypes AS136 and AS135 with mean values of 92 and 71 days, and 149 and 151 days in that order. Plant height varied from 107.06 to 223.28 cm. The mean plant height for the evaluated genotypes was 156.01 cm. The tallest genotypes with mean plant height greater than 180 cm were AS205, AS391, AS109, AS113, and AS111. The shortest genotype across the testing locations was PAN8816. The mean total plant biomass of the evaluated genotypes was 27.94 g plant^-1^. The total plant biomass of the genotypes ranged from 14.07 to 43.75 g plant^-1^, with genotypes AS122, AS391, SS27, AS203, AS74 having the highest total plant biomass of 43.75, 41.31, 38.65, 38.19, 37.83 g plant^-1^, respectively. The mean shoot biomass varied from 6.49 to 24.87 g plant^-1^, with a grand mean of 16.31 g plant^-1^. The most productive genotypes with the highest shoot biomass were SS27, AS122, AS203, and AS391, with 24.87, 23.90, 23.45, and 23.27 g plant^-1^, respectively. The grand mean root biomass for the evaluated genotypes was 11.62 g plant^-1^, ranging from 5.92 to 21.02 g plant^-1^. The highest shoot biomass was 21.02, 20.31, 19.87, 19.85, 19.46, and 18.04 g plant^-1^ observed on genotypes AS106, AS74, AS72, AS122, and AS152. The root-to-shoot biomass ratio ranged from 0.32 to 3.00. The genotypes that allocated more biomass to their roots than their shoots were AS152, AS106, and 05-POTCH-138, with the highest root-to-shoot biomass ratio of 3.00, 2.50, and 1.95, respectively. The wide genetic variation in grain yield spanned from 2.53 to 25.08 g plant^-1^, averaging 11.90 g plant^-1^. Genotypes AS115, AS251, AS134, AS145, and AS130 were the five best-performing genotypes with mean yields of 25.08, 21.83, 21.42, 19.43, 18.50 g plant^-1^, respectively. The harvest index ranged from 15.34 to 66.66%. Genotypes AS115, AS130, and AS251 exhibited the highest harvest index ≥ 60%.

**Table 3:** Mean values for the agronomic traits among the ten best and five bottom genotypes after evaluating 50 sorghum genotypes across three locations.

Genotype	DTH	DTM	PH	PB	SB	RB	RS	GY	HI
Top ten genotypes									
AS115	88	130	182.50	21.39	12.54	8.85	0.71	25.08	66.66
AS251	85	142	131.39	27.60	14.63	12.97	0.89	21.83	59.88
AS134	78	135	162.72	35.52	20.37	15.16	0.74	21.42	51.26
AS145	86	141	157.67	32.20	21.95	10.25	0.47	19.43	46.95
AS130	77	145	163.33	25.56	11.29	14.27	1.26	18.50	62.09
SS27	79	142	135.92	38.65	24.87	13.77	0.55	17.58	41.41
AS138	78	140	146.17	28.54	15.03	13.51	0.90	17.45	53.73
AS132	94	133	171.06	25.90	15.61	10.29	0.66	16.89	51.98
AS563	84	135	173.94	33.02	21.31	11.71	0.55	16.83	44.12
AS203	82	131	174.50	38.19	23.45	14.74	0.63	16.63	41.49
Bottom five genotypes									
AS147	80	138	145.22	16.20	7.51	8.69	1.16	4.63	38.13
AS116	77	138	142.11	21.52	14.89	6.63	0.45	4.35	22.62
PAN8816	75	140	107.06	33.58	17.47	16.12	0.92	3.75	17.68
AS129	84	143	132.28	26.89	17.41	9.49	0.54	3.28	15.87
AS111	84	144	185.67	23.22	13.97	9.25	0.66	2.53	15.34
Mean	79.52	138.01	156.01	27.94	16.31	11.62	0.8	11.9	41.47
SD	6.82	5	22.74	6.56	4.74	4.26	0.52	5.03	12.09
SE	0.97	0.71	3.22	0.93	0.67	0.6	0.07	0.71	1.71
Skewness	0.5	0.38	0.47	0.23	− 0.26	0.6	2.48	0.25	− 0.34
kurtosis	0.22	0.09	0.4	− 0.25	− 0.73	− 0.55	7.41	− 0.13	− 0.3

SD = standard deviation, SE = standard error, DTH = days to 50% heading, DTM = days to 50% maturity, PH = plant height (cm), PB = total plant biomass (g plant^-1^), SB = shoot biomass (g plant^-1^), RB = root biomass (g plant^-1^), RS = root to shoot biomass ratio, GY = grain yield (g plant^-1^), HI = harvest index (%).

#### Carbon allocation to roots and shoots

The mean performance of the 25 selected sorghum genotypes for carbon storage (SCc, RCc, GCc, PCs, SCs, RCs, RCs/SCs, and GCc) is summarized in Table 4. The top ten genotypes, based on their high root carbon stock, are highlighted in bold fonts. All the carbon content variables ranged from 40 to 45%. The total plant carbon stocks ranged from 7.52 to 24.64 g plant^-1^, with a mean of 12.65 g plant^-1^. The genotypes that sequestered more carbon with the highest total plant carbon stock were SS27, AS122, AS134, AS203, and AS563, with values of 24.64, 18.00, 16.48, 15.55, and 14.99 g plant^-1^, respectively. The lowest carbon sequestration with the lowest total plant carbon stock of 7.96, 7.69, and 7.52 g plant^-1^ was recorded in genotypes NW5393, AS116, and AS115, respectively. The shoot carbon stock of the selected genotypes had a mean of 7.98 g plant^-1^, spanning from 3.25 to 19.04 g plant^-1^. The genotypes that allocated more carbon to the shoots with the highest shoot carbon stock were SS27, AS122, ICSV92001, and AS563 with 19.04, 10.42, 10.34, and 10.33 g plant^-1^, respectively. The root carbon stocks varied from 1.38 to 8.37 g plant^-1^. The mean root carbon stock for the selected genotypes was 4.67 g plant^-1^. The genotypes that had the highest root carbon stock were AS108 with 8.87 g plant^-1^, followed by AS122 (7.58 g plant^-1^), AS134 (7.13 g plant^-1^), AS251 (6.49 g plant^-1^), and AS203 (6.40 g plant^-1^). Genotypes AS145 and AS116 were among the genotypes with the lowest root carbon stock with 1.74 and 1.37 g plant^-1^, respectively. The root-to-shoot carbon stock ratio ranged from 0.18 to 1.56. The genotypes allocated more carbon to their shoots than their roots. The genotypes that allocated more carbon to their roots than their shoots were AS108 and AS115, with the highest root-to-shoot carbon stock ratio of 1.56 and 1.31, respectively. The grain carbon stocks ranged from 1.04 to 12.92 g plant^-1^, with a grand mean of 5.84 g plant^-1^. The genotypes AS115 and AS134 were the highest grain carbon stock, with mean grain carbon stock of 12.92 and 11.38 g plant^-1^, respectively.

**Table 4:** Mean values for carbon storage traits of the 25 selected sorghum genotypes evaluated during the 2022 growing season at Silverton, South Africa.

Genotype	SCc	RCc	GCc	PCs	SCs	RCs	RCs/SCs	GCs
**16MZ**	**43.20**	**44.41**	**43.24**	**14.92**	**9.83**	**5.09**	**0.52**	**5.24**
**AS108**	**43.74**	**44.79**	**43.96**	**14.55**	**5.68**	**8.87**	**1.56**	**3.92**
AS109	43.95	42.53	43.06	11.40	6.47	4.93	0.76	9.40
**AS111**	**43.91**	**45.40**	**43.64**	**12.86**	**7.40**	**5.46**	**0.74**	**1.04**
AS115	43.44	45.34	43.56	7.52	3.25	4.27	1.31	12.92
AS116	43.78	40.12	43.34	7.69	6.33	1.37	0.22	1.78
AS117	43.51	38.33	43.51	12.23	9.13	3.09	0.34	4.91
**AS122**	**43.62**	**40.39**	**43.43**	**18.00**	**10.42**	**7.58**	**0.73**	**3.69**
AS130	43.73	40.55	43.40	8.65	4.33	4.32	1.00	9.26
AS131	43.51	41.76	44.06	13.15	8.76	4.39	0.50	3.38
AS132	44.48	38.55	43.16	13.77	9.20	4.57	0.50	9.31
**AS134**	**44.41**	**39.34**	**43.25**	**16.48**	**9.34**	**7.13**	**0.76**	**11.38**
AS136	44.17	39.15	43.58	9.00	4.97	4.03	0.81	6.57
AS138	44.87	40.72	43.36	8.52	5.35	3.17	0.59	8.04
AS143	44.68	43.67	44.24	8.16	5.41	2.76	0.51	3.87
AS145	45.16	33.69	43.64	11.64	9.90	1.74	0.18	6.68
**AS203**	**44.56**	**43.41**	**43.47**	**15.55**	**9.15**	**6.40**	**0.70**	**7.23**
**AS251**	**43.51**	**43.26**	**42.89**	**14.97**	**8.48**	**6.49**	**0.76**	**4.57**
AS563	44.70	40.26	44.49	14.99	10.33	4.66	0.45	5.91
ICSV92001	44.55	39.94	44.65	14.33	10.34	3.99	0.39	3.35
**LP4403**	**43.73**	**43.76**	**45.31**	**13.89**	**8.38**	**5.51**	**0.66**	**3.35**
MAMOLOKWAN	44.35	41.17	43.44	8.94	5.47	3.47	0.64	5.33
NW5393	45.09	39.40	44.07	7.96	5.20	2.75	0.53	5.73
**PAN8816**	**44.44**	**37.71**	**43.24**	**12.54**	**7.38**	**5.16**	**0.70**	**1.62**
**SS27**	**45.48**	**39.92**	**43.27**	**24.64**	**19.04**	**5.61**	**0.29**	**7.61**
Mean	44.18	41.10	43.65	12.65	7.98	4.67	0.65	5.84
SD	0.61	2.76	0.56	3.96	3.14	1.77	0.31	3.02
SE	0.12	0.55	0.11	0.79	0.63	0.35	0.06	0.60
Skewness	0.35	− 0.44	1.42	0.96	1.66	0.34	1.25	0.56
Kurtosis	− 0.81	0.64	2.10	2.01	5.41	0.24	2.50	− 0.07

SD = standard deviation, SE = standard error, SCc = shoot carbon content (%), RCc = root carbon content (%), GCc = grain carbon content (%), PCs = total plant carbon stocks (g plant^-1^), SCs = shoot carbon stock (g plant^-1^), RCs = root carbon stock (g plant^-1^), RCs/SCs = root to shoot carbon stock ratio, GCs = grain carbon stock (g plant^-1^). The top ten performing genotypes based on RCs are highlighted in bold fonts.

#### Principal component and biplot analyses for agronomic traits

Table 5 displays the rotated component matrix, illustrating the percentage variance associated with various principal components (PCs) and the corresponding loadings for recorded agronomic traits. Four principal components (PC1 to PC4) attributed to 86.21% of the total genotypic variation for agronomic- and biomass-related traits. Total plant biomass and SB made the highest contributions to PC1, followed by RB and RS, with positive contributions to PC2. The highest positive loadings for PC3 were for PH and DTM, and for PC4 were for GY and HI, respectively.

**Table 5:** Principal components showing variation and contribution by nine agronomic traits among 50 sorghum genotypes evaluated during the 2022 and 2023 growing seasons at three locations in South Africa.

Trait	PC1	PC2	PC3	PC4
DTH	0.03	− 0.21	**0.66**	0.13
DTM	− 0.01	− 0.28	− 0.74	0.003
PH	0.09	− 0.05	**0.88**	0.04
PB	**0.94**	0.34	0.08	0.02
SB	**0.93**	− 0.32	0.09	− 0.05
RB	**0.41**	**0.89**	0.02	0.09
RS	− 0.34	**0.92**	− 0.05	0.05
GY	0.23	− 0.06	0.15	**0.95**
HI	− 0.36	0.25	0.08	**0.89**
Eigenvalue	2.37	2.27	1.84	1.29
Variance (%)	26.29	25.17	20.39	14.36
Cumulative variance (%)	26.29	51.46	71.85	86.21

PC = principal component, DTH = days to 50% heading, DTM = days to 50% maturity, PH = plant height, PB = total plant biomass, SB = shoot biomass, RB = root biomass, RS = root to shoot biomass ratio, GY = grain yield, HI = harvest index. The highest loading scores for each PC are highlighted in bold.

**Figure 1:**
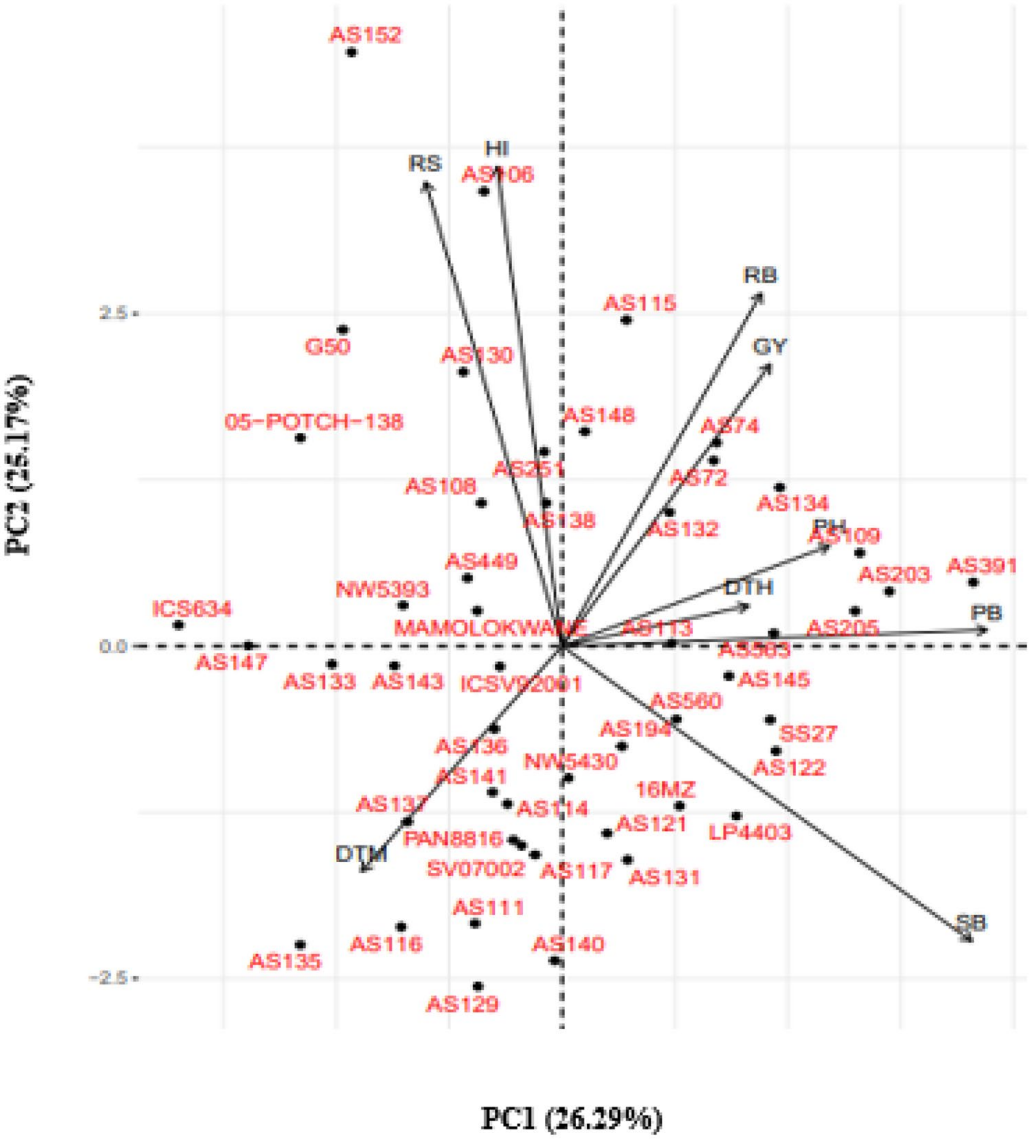
Principal component biplot displaying the relationship among nine agronomic traits of 50 sorghum genotypes evaluated in three locations in South Africa. PC1 = first principal component, PC2 = second principal component, DTH = days to 50% heading, DTM = days to 50% maturity, PH = plant height, PB = total plant biomass, SB = shoot biomass, RB = root biomass, RS = root to shoot biomass ratio, GY = grain yield, HI = Harvest index.

Biplot generated through principal component analysis for agronomic traits is illustrated in Fig. 1. The first principal component (PC1) was positively correlated with DTH, PH, PB, SB, RB, and GY. On the contrary, PC2 was negatively correlated with DTM, RS, and HI. Agronomic traits like DTH, PH, PB, SB, RB, and GY were positively associated with each other, evident in their vectors aligning in the same direction and forming acute angles between them (Fig. 1). Similarly, DTM, RS, and HI were positively correlated to each other. High-yielding genotypes such as AS134 and SS27 had high PB and SB, while AS115 and AS251 were associated with RB, HI, and RS (Fig. 1).

#### Principal component and biplot analyses for carbon storage

The PCA showed that three PCs accounted for 78.18% of the total variation. The first component had the major contribution of 32.68% to the variation (Table 6). Variation in PC1 was mainly from the positive loadings of RCs/SCs, RCc, and RCs and negative loadings of SCc. The second principal component (PC2) contributed 27.68% to the variation, mainly from the positive contributions of PCs, SCs, and RCs. The variation in PC3 (17.83%) was due to positive GCs and RCs/SCs loadings, and the negative loadings were due to GCc and RCc.

**Table 6:** Principal components showing variation and contribution by carbon storage among 25 selected sorghum genotypes.

Trait	PC1	PC2	PC3
SCc	− 0.72	0.15	0.12
RCc	**0.83**	0.01	− 0.18
GCc	0.03	− 0.07	− 0.75
PCs	− 0.02	**0.98**	0.01
SCs	− 0.40	**0.89**	− 0.07
RCs	**0.67**	**0.66**	0.15
RCs/SCs	**0.86**	− 0.12	**0.31**
GCs	0.01	− 0.07	**0.85**
Eigenvalue	2.61	2.21	1.43
Variance (%)	32.68	27.68	17.83
Cumulative variance (%)	32.68	60.35	78.18

PC = principal component, SCc = shoot carbon content, RCc = root carbon content, GCc = grain carbon content, PCs = total plant carbon stocks, SCs = shoot carbon stock, RCs = root carbon stock, RCs/SCs = root to shoot carbon stock ratio, GCs = grain carbon stock. The highest loading scores for each PC are highlighted in bold.

**Figure 2:**
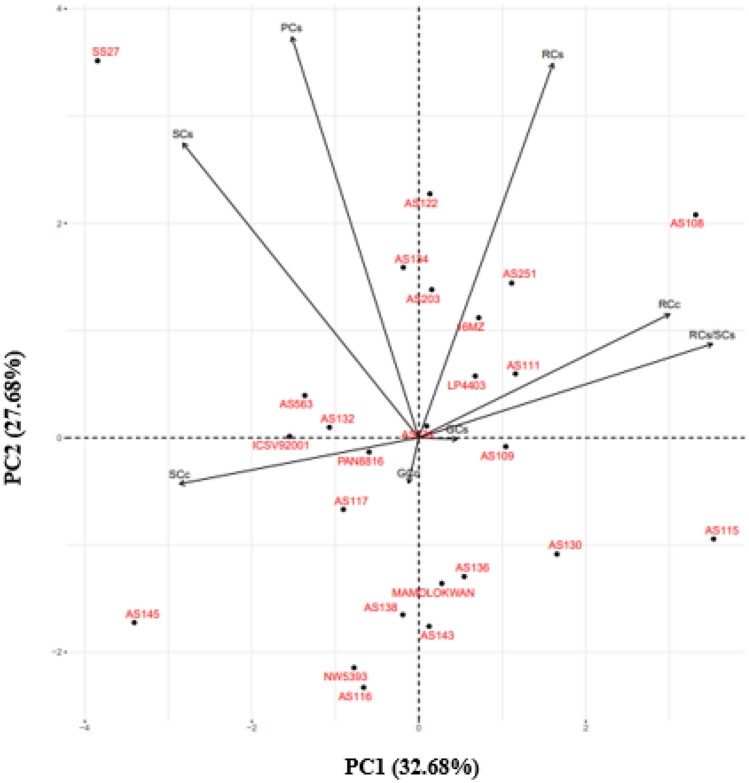
Principal component biplot displaying the relationship among carbon storage traits in the 25 selected sorghum genotypes. PC1 = first principal component, PC2 = second principal component, SCc = shoot carbon content, RCc = root carbon content, GCc = grain carbon content, PCs = total plant carbon stocks, SCs = shoot carbon stock, RCs = root carbon stock, RCs/SCs = root to shoot carbon stock ratio, GCs = grain carbon stock.

Characters such as RCc, PCs, SCs, RCs, RCs/SCs, and GCs were positively correlated. Negative correlations were observed between SCc, RCc, GCc, RCs, and RCs/SCs (Fig. 2). A strong positive correlation was observed between RCc, RCs/SCs, and GCs. There was a strong correlation between PCs and SCs. A strong negative correlation was observed between GCc and RCs. The genotypes were equally scattered across both PC1 and PC2. Genotypes SS27, LP4403, and AS111 scored higher values for SCs, PCs, and RCs. Genotypes PAN8816 and ICSV92001 had a strong association with SCc.

#### Principal component and biplot analyses for agronomic traits and carbon storage

The principal component analysis was performed to identify the most discriminative variables among the sorghum genotypes. A total of 82.51% of the variation explained by the agronomic traits and carbon storage traits were explained by the five principal components (Table 7). Generally, traits such as PB, PCs, SB, SCs, RCs/SCs, RS, GY, and GCc contributed much to the variations in the PCs. Nevertheless, PB, PCs, SB, SCs, and RB, had the highest contributions (with contributions of 0.90, 0.89, 0.82, 0.81, and 0.67, respectively) to PC1. Traits like RCs/SCs, RS, and RCs were highest (0.86, 0.78, and 0.70, respectively) positive contributors in PC2. The traits that contributed the most in PC3 were GY and GCs (0.78 and 0.74). The fourth principal component accounted for 76.43% of the total variation, with PH and Rcc (0.72 and 0.59) exhibiting the highest positive loadings for PC4. GCc was the only positive contributor to the observed variation on PC5 with a PC loading of 0.68.

**Table 7:** Principal components showing variation and contribution by agronomic traits and carbon storage traits among 25 selected sorghum genotypes.

Trait	PC1	PC2	PC3	PC4	PC5
DTH	− 0.32	0.23	0.39	0.17	− 0.25
DTM	0.18	− 0.10	− 0.35	− 0.66	0.02
PH	− 0.47	0.07	0.25	**0.72**	0.08
PB	**0.90**	0.23	0.16	0.12	− 0.09
SB	**0.82**	− 0.17	0.32	0.27	− 0.18
RB	**0.67**	0.64	− 0.10	− 0.12	0.07
RS	− 0.01	**0.78**	− 0.35	− 0.36	0.24
GY	− 0.15	0.47	**0.78**	− 0.13	0.04
HI	− 0.48	0.48	0.58	− 0.23	0.20
SCc	0.06	− 0.30	0.55	− 0.24	0.54
RCc	− 0.17	0.43	− 0.44	**0.59**	− 0.02
GCc	− 0.09	− 0.31	− 0.22	0.43	**0.68**
PCs	**0.89**	0.12	0.18	0.15	0.13
SCs	**0.81**	− 0.25	0.37	0.07	0.10
RCs	0.57	**0.70**	− 0.24	0.20	0.11
RCs/SCs	− 0.20	**0.86**	− 0.36	0.06	0.08
GCs	− 0.23	0.54	**0.74**	0.03	− 0.09
Eigenvalue	4.44	3.61	3.00	1.95	1.03
Variance (%)	26.11	21.23	17.65	11.45	6.08
Cumulative variance (%)	26.11	47.33	64.98	76.43	82.51

PC = principal component, DTH = days to 50% heading, DTM = days to 50% maturity, PH = plant height, PB = total plant biomass, SB = shoot biomass, RB = root biomass, RS = root to shoot biomass ratio, GY = grain yield, HI = harvest index, SCc = shoot carbon content, RCc = root carbon content, GCc = grain carbon content, PCs = total plant carbon stocks, SCs = shoot carbon stock, RCs = root carbon stock, RCs/SCs = root to shoot carbon stock ratio, GCs = grain carbon stock. The highest loading scores for each PC are highlighted in bold.

**Figure 3:**
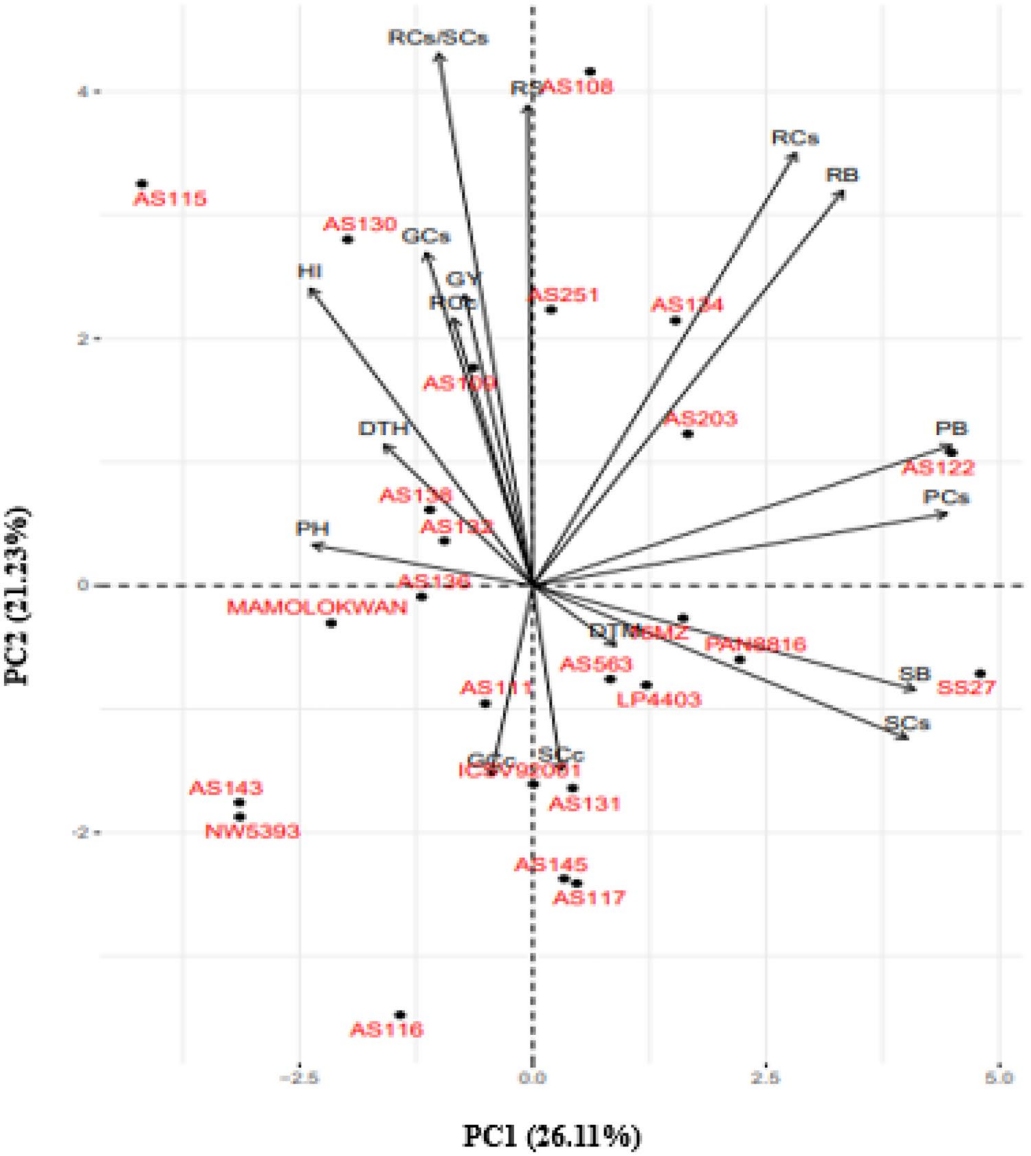
Principal component biplot displaying the relationship among agronomic traits and carbon storage traits in the 25 selected sorghum genotypes. PC1 = first principal component, PC2 = second principal component, DTH = days to 50% heading, DTM = days to 50% maturity, PH = plant height, PB = total plant biomass, SB = shoot biomass, RB = root biomass, RS = root to shoot biomass ratio, GY = grain yield, HI = Harvest index, SCc = shoot carbon content, RCc = root carbon content, GCc = grain carbon content, PCs = total plant carbon stocks, SCs = shoot carbon stock, RCs = root carbon stock, RCs/SCs = root to shoot carbon stock ratio, GCs = grain carbon stock.

Biplots based on the principal component analysis were drawn for agronomic traits and carbon storage traits (Fig. 3). High yielding and early flowering genotypes such as AS115, AS132, and AS203 had high GCs, RCc, RS, and RCs/SCs, while 16MZ, PAN8816, and SS27 were associated with SB and SCs. Grain yield had strong correlation with GCs, RCs, RS, RCs/SCs, RB, PB, and high yielding genotypes including AS138 and AS130. Shoot biomass was highly correlated with SCs and high carbon storage genotypes including SS27, 16MZ, and AS563.

#### Cluster analysis for agronomic traits

**Figure 4:**
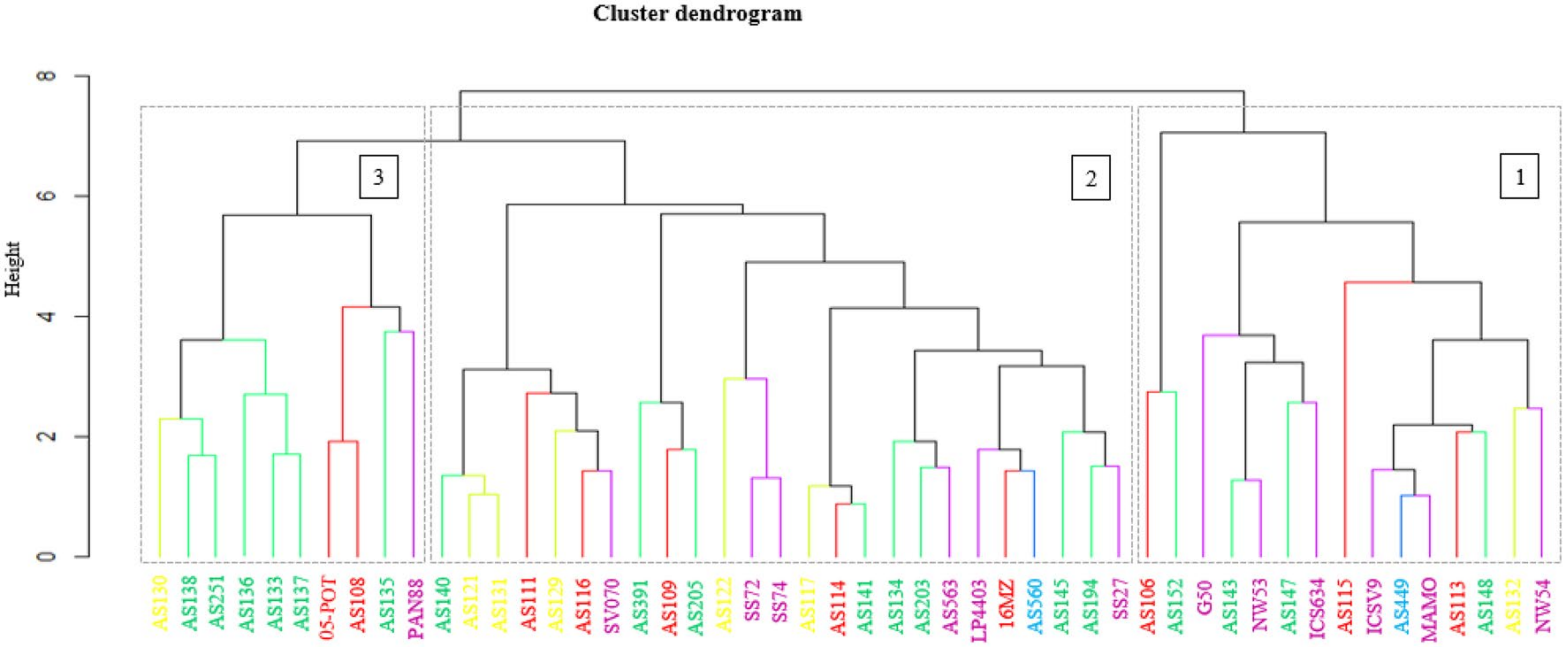
Hierarchical cluster dendrogram based on agronomic traits showing genetic similarity matrix of 50 sorghum genotypes evaluated in three locations in South Africa.

The assessment of the phenotypic diversity using agronomic traits delineated the genotypes into three distinct clusters (Fig. 4). The second cluster had the highest number (25) of genotypes, while the first cluster had 15, and the third cluster had 10 genotypes. Nevertheless, all three clusters comprised a combination of landraces, breeding lines, cultivars, and origins in their genotype composition.

#### Cluster analysis for carbon storage

**Figure 5:**
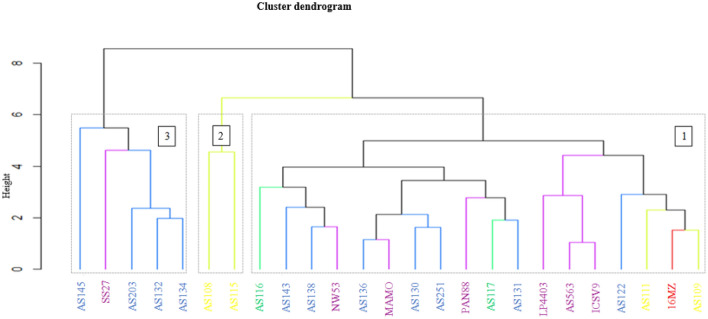
Hierarchical cluster dendrogram based on carbon storage traits showing genetic similarity matrix of the 25 selected sorghum genotypes.

The results of cluster analysis for carbon storage are presented in Fig. 5. Genotypes AS109, 16MZ, AS111, AS122, ICSV92001, AS563, LP4403, AS131, AS117, PAN8816, AS251, AS130, MAMOLOKWANE, AS136, NW5393, AS138, AS143, and AS116 were grouped together in the first cluster. The second cluster consisted of genotypes AS115 and AS108. The third cluster included genotypes AS134, AS132, AS203, SS27, and AS145.

## Discussion

The current study assessed 50 sorghum genotypes across three different locations for their performance in terms of growth, yield stability, and adaptability, with the aim of identifying key genetic traits that contribute to resilience and productivity under diverse environmental conditions. A combined analysis of variance revealed that genotypes showed significant variation in agronomic traits, indicating the presence of marked genetic variability in developing new sorghum cultivars with high grain and biomass yields^30^. Mulima^31^ reported significant variations in agronomic traits among sorghum genotypes obtained from the International Crops Research Institute for the Semi-Arid Tropics (ICRISAT) gene bank. Genotype performances were also impacted by significant genotype x location interactions, indicating that the performance of genotypes varied across different environments. Enyew^32^ reported a significant genotype x environment (G x E) interaction on yield and yield-components of sorghum genotypes. Environmental influences play a significant role in phenotypic variations, and the differential responses of genotypes to environmental conditions contribute to the observed variability^33^. Understanding the effect of G x E and predicting the phenotypic response to various environments are vital to improving the selection efficiency in sorghum breeding programs. Phenotypic expression provides a critical screening and breeding method to exploit genetic variability. The significant variation observed in biomass and grain yield production among genotypes could be attributed to water availability, temperature, humidity, and soil fertility^34^. Furthermore, variation in agronomic performances may be influenced by the amount and distribution of rainfall, leading to adequate moisture and favorable temperature during panicle development and flowering. Low CVs were recorded for DTM, PB, RB, GY, and HI, indicating that these traits could be prioritized for assessing sorghum genotypes.

The significant differences among the sorghum genotypes for GCc, PCs, SCs, RCs, RCs/SCs, and GCs, indicate the availability of sufficient genetic diversity in the test genotypes for carbon sequestration. Related results have been reported in sorghum genotypes^25,35^. Genotypes that exhibit a wide range of trait values may be better equipped to thrive in diverse growing conditions^36^.

The higher grain yield was recorded for genotypes SS27, AS203, AS145, AS563, and AS134 possibly due to their higher number of grains per head, likely influenced by their high shoot biomass. This aligns with the findings of George-Jaeggli^37^ which emphasized that seed number is the most crucial yield component associated with increases in sorghum yields. The genotypes AS115, AS251, AS203, and AS138 consistently produced the highest yields in all three environments, suggesting the stable performance of the genotypes in diverse growing environments. The four genotypes expressed tall plant stature across the environments. These findings are consistent with the results reported by George-Jaeggli^37^, where increased plant height positively affected grain yield via an effect on shoot biomass. High-shoot biomass increases grain production through increasing leaf area for light absorption and carbon assimilation to facilitate grain filling^38^. Breeders can select these genotypes as they have demonstrated to be less sensitive to changes in environmental conditions. They can maintain reasonable yields even in adverse conditions, such as excessive rainfall or drought^39^. Drought stress affects the photosynthetic rate of sorghum through various physiological mechanisms including reduced stomatal conductance and transpiration rate, lowered quantum yield, increased leaf temperature, decreased chlorophyll content and ribulose-1,5-bisphosphate carboxylase, increased oxygen evolution, and reduced phosphoenolpyruvate photosynthesis activity^40^. Previous findings indicated that drought-tolerant sorghum genotypes exhibit a significant increase in chlorophyll fluorescence and photosynthetic rate under drought stress conditions^41,42^. Getnet^43^ reported that drought tolerant sorghum genotypes exhibit an increased photosynthetic rate, supplying the required raw material and energy for growth and development, enabling them to sustain their grain yield under drought stress. Genotypes AS74 and AS72 ranked second and third for RB production, respectively, and these genotypes were among the genotypes with slightly high GY. These results are supported by the strong association between root biomass and grain yield, demonstrating the significance of root traits in enhancing productivity. Increased root growth improves plant capacity and efficiency in acquiring nutrients and moisture and enhancing agro-ecosystem resilience^30^. This is particularly vital under drought conditions when there is less water in the soil profile, and a deeper and larger root system can forage for water^44^. Though larger root systems in crops are beneficial, particularly in arid areas, they may be inefficient or even result in a production penalty in wet seasons or regions with enough water and capacity to provide additional irrigation^45^. However, evidence from this study suggests that root biomass positively affects productivity. These results are consistent with the ones reported by Fang^46^, who pinpointed the contribution of deeper and more profuse root growth to grain production. These larger root diameters can be used to boost soil carbon in agricultural soils. Identification of genotypes based on biomass production and allocation will allow for a more effective explanation of differences between individual genotypes^47^. The harvest index (HI) indicates the proportion of grain production to above-ground plant biomass. HI plays a vital role in selecting high-yielding genotypes. A high HI reflects a genotype’s ability to efficiently convert biological yield into economic yield^48^. Genotypes AS111 and AS147 had the lowest GY due to their low biomass allocation to the roots and shoots, respectively. Genotype AS111 had the lowest HI of 4.84%. Preventing losses in grain production per plant requires a combination of minimal biomass reduction and an increased HI^37^. Grain yield formation in sorghum is often sink-limited rather than source-limited^49^.

Genotypes NW5393, AS116, and AS115 sequestered less carbon as they accumulated the lowest total plant carbon stocks. This was associated with their low production of biomass. Genotypes SS27, AS122, AS134, AS203, and AS563 had high biomass production, which increased their capacity to sequester more C^50^. All test genotypes stored more carbon in their shoots, indicating that roots are weaker C sinks than shoots. Because C is only exported to other sinks when supply exceeds local demand, SCs are higher than RCs. A wide range was recorded among the test genotypes for root biomass. This variation can be exploited in breeding sorghum genotypes for productivity and carbon sequestration. Root biomass could be an accurate indicator of crop C intake into the soil^51^. Despite sorghum genotypes in the current study allocating less C into their root system than the shoot, certain sorghum genotypes developed more root volume, increasing their competitiveness for nutrients^52^. Root-to-shoot biomass ratio (RS) and root-to-shoot carbon stock ratio (RCs/SCs) increased as PB and PCs decreased, respectively. This might be due to increased root biomass regulated by growth hormones like trans-zeatin riboside. Andreas^53^ reported that cytokinins played an important role in plant growth. These results aligned with the study conducted by Qi^54^, who reported a negative correlation between RS and PB. Brassard^55^ reported that the RCs/SCs regulates carbon partitioning within shoots and roots.

Increased carbon allocation to the roots, higher root-to-shoot ratios may result in significantly less carbon storage in above-ground biomass. When a plant allocates more carbon to its roots, it invests a larger proportion of its resources below ground^56^. Due to the sorghum genotypes genetic make-up, different genotypes may exhibit different RS and RCs/SCs. Genotypes AS108, AS115, and AS130 might have evolved to allocate C more efficiently to their roots, influencing C storage patterns^57^. Genotype AS108 ranked fifth and first for exhibiting the highest RS and RCs/SCs, respectively, but relatively producing low yields, these results are supported by Larson^58^, who also reported that if the trade-off oscillates too much towards root development, an excessively high RS may result in diminished above-ground growth and, eventually, lower grain yields. An increase in total plant biomass production was associated with high grain yield, it can be concluded that instead of changing RS; increasing PB can increase yield without reducing root carbon sequestration capability. It is feasible to increase root and shoot biomass simultaneously to attain high PB, as demonstrated by the balanced production of biomass in genotype AS251. This study confirmed that most sorghum genotypes can be C sink in soils. The amount of C stock in soil by several sorghum genotypes is affected by land-use change and sorghum management practices^59^.

The principal component analysis facilitated the recognition of significant agronomic traits that displayed substantial variability among the evaluated genotypes. The present study identified PB, SB, RB, RS, and GY as the most crucial traits, given their substantial contributions to PC1 and PC2. Abraha^29^ and Mangena^28^ confirmed the significance of grain yield, dry matter, and biomass to sorghum improvement. Furthermore, the analysis of principal components revealed that the diversity observed in the test genotypes cannot be fully explained (accounts for 86.21% of the total variation) by a limited set of characteristics. This suggests that numerous traits explain the overall variance among the accessions. In descending order of significance DTM, DTH, PH, RS, and HI were identified as the major contributors to explaining a substantial proportion of the entire phenotypic diversity. These findings are confirmed by Ayana and Bekele^60^, who reported these traits in contributing to the overall diversity of sorghum landraces.

The current study identified RCs/Scs, RCc, RCs, PCs, and SCs as the most important traits, given their significant contribution to both PC1 and PC2. These findings suggest the importance of these traits for selection. Accessions exhibiting increased, and desirable mean performances in these targeted traits would be selected for further enhancement. The genotypes were equally scattered across both PC1 and PC2, this indicates that there is an even distribution of genotypic characteristics along the associated axes. This could suggest that the genetic variations shown by both components are not contributing significantly to the observed variation among genotypes. Maximum weight should be given to traits with strong positive loadings, notably GCs, in the third component. According to Upadhyaya^61^ and Soroj^62^, trait contribution to various PCs differs with genetic diversity within the assessed germplasm and the number of traits evaluated. The current study suggested that the above traits could play a significant role in atmospheric carbon sequestration.

The cluster analysis outlined the genotypes into three distinct groups, containing significantly different numbers of genotypes. The cluster analysis was able to group the genotypes based on flowering period. The first cluster consists of early flowering genotypes, the second of intermediate maturing genotypes, and the third of late flowering genotypes. This analysis demonstrated that genotype information on the flowering period may be relevant in identifying parents with various maturing groups^31^. The traits PB, SB, RB, and GY are most distinguished between the clusters. Grouping the genotypes by traits may reveal that the genotypes are similar in one or more traits. Promising genotypes can be found using cluster means of assessed traits^63^. Billot^64^ reported that the breeder must better understand the genetics of agronomic traits to maximize the efficiency of selecting more diverse and suited parents for cultivar development.

The grouping of genotypes into distinct clusters highlights the diversity in the pedigree of the test genotypes, as these clusters represent relatedness within the genetic lineage of the genotypes^65^. The analysis suggests a high level of genetic diversity among genotypes for carbon storage. The genotypic variation present in the germplasm provides potential for sorghum improvement by selecting the best performing genotypes from various clusters to retain genetic diversity, which is essential for breeding^66^.

## Conclusion

The genotypes displayed varying agronomic traits and carbon sequestration capacities, which can be exploited in breeding sorghum genotypes with high productivity and carbon sequestration potential. Some genotypes accumulated more carbon in their biomass, implying their high capacity of the genotypes to absorb more carbon from the atmosphere. Deeper and larger roots are important to improve carbon sequestration, soil fertility, and crop productivity. Further, the study found a high root-to-shoot ratio of carbon as a priority trait in estimating carbon sequestration capacity in sorghum. Overall, genotypes such as AS251, SS27, AS134, AS203, and AS563 were selected for their high biomass production, grain yield, and C sequestration potentials. The selected sorghum genotypes are recommended for production or further breeding and variety release adapted to various agroecologies in South Africa. Selecting genotypes with high C storage ability is a practical strategy for climate change mitigation, as it reduces land degradation. This approach also ensures soil health and sustainable productivity, thereby addressing the issue of food insecurity.

## Materials and methods

### Plant materials

Fifty sorghum genotypes consisting of landraces, pure lines and commercial hybrids were used in this study (Table 8). The test germplasms were obtained from different sources, including Zimbabwe, South Africa, Ethiopia, and Tanzania. South African genotypes were collected from KwaZulu-Natal, Eastern Cape, and Limpopo Provinces and mainlined at the African Centre for Crop Improvement (ACCI) of the University of KwaZulu-Natal (UKZN) in South Africa. The genotypes were selected for their high grain yield, biomass, and ethanol production^28^.

**Table 8:** Detailed description of sorghum genotypes used in this study.

Name	Pedigree	Source	Seed colour	Country	Name	Pedigree	Source	Seed colour	Country
05-POTCH-138	50-POTCH-138	ARC-GCI	White	SA	AS143	Red Swazi	ACCI	Brown	SA
16MZ	–	–	Brown	–	AS145	AWN98	ACCI	Brown	SA
AS106	Landrace	ACCI	Cream	SA	AS147	MRS94	ACCI	Red	SA
AS108	P9504B	ACCI	Cream	SA	AS148	SDS 3472	ACCI	Brown	SA
AS109	P9511B	ACCI	Cream	SA	AS152	01MN1589	ACCI	Brown	SA
AS111	P9539B	ACCI	Cream	SA	AS194	Mtentu Imphe	D Vatcha	Brown	–
AS113	TX2737/91BE7414	ACCI	Cream	SA	AS203	SA landrace LP 49	J M Donaldson	Brown	SA
AS114	BTx3197	ACCI	Cream	SA	AS205	SA landrace LP 51	J M Donaldson	Brown	SA
AS115	BTx631	ACCI	Cream	SA	AS251	AS97 OPV	ACCI	Red	SA
AS116	01Aphid207	ACCI	Cream	SA	AS391	SS27 OPV	Mtentu	Brown	SA
AS117	01Aphid148	ACCI	Cream	SA	AS449	#12235926 OC	Ethiopia	Red	Ethiopia
AS121	Kat 369 × EX-1 Chira	ACCI	Brown	SA	AS560	IESV 92028 DL	ICRISAT	Brown	–
AS122	KSV 12	ACCI	Cream	SA	AS563	IS 2331	ICRISAT	Brown	–
AS129	KARI Mtama X ICS 3-1	ACCI	Cream	SA	AS72	KAT-487	UK-SGVT 07-49	Cream	–
AS130	Gambella 1107	ACCI	Cream	SA	AS74	ICSV 111	UK-SGVT 07-51	Brown	–
AS131	WK#1025 Sudan	ACCI	Cream	SA	G50	TZA 5557	Tanzania	Brown	Tanzania
AS132	Parc 1260793	ACCI	Cream	SA	ICS634	–	ICRISAT	Brown	–
AS133	Marimanti Co 1110	ACCI	Cream	SA	ICSV92001	–	ICRISAT	Brown	–
AS134	P6 NQ#23 Sudan	ACCI	Brown	SA	LP4403	LP4403	ARC-GCI	Brown	SA
AS135	Dinkmash	ACCI	Cream	SA	MAMOLOKWANE	Mamolokwane	ARC-GCI	White	SA
AS136	FLO (107) x GS 3541	ACCI	Cream	SA	NW5393	–	ARC-GCI	Brown	SA
AS137	IESV 92022 DL	ACCI	Grey	SA	NW5430	–	ARC-GCI	Brown	SA
AS138	Mugeta	ACCI	White	SA	PAN8816	PAN8816	Pannar	Red	SA
AS140	Kaguru	ACCI	Red	SA	SS27	SS27	ARC	Brown	SA
AS141	Kiboko loca	ACCI	Red	SA	SV07002	–	ICRISAT	Brown	–

ARC-GCI = Agricultural Research Council–Grain Crops Institute, ACCI = African Centre for Crop Improvement, UKZN = University of KwaZulu-Natal, ICRISAT = International Crops Research Institute for the Semi-Arid Tropics, SA = South Africa, – = unknown.

### Study sites

Field experiments were conducted during the 2022 and 2023 growing seasons at three South African locations (Silverton, Ukulinga, and Bethlehem). The Silverton location is the main research station of the Agricultural Research Council–Agricultural Engineering, located on the outskirts of Pretoria (latitude: 25°44' S, longitude: 28°14' E). The mean annual temperature and rainfall for the location was 18.4 ℃ and 661 mm, respectively. The Ukulinga Research location is located at Farm of the University of KwaZulu-Natal in Pietermaritzburg (latitude: 30°24' S, longitude: 29°24 E'). The long-term average temperature and rainfall for Ukulinga are 16.7 ℃ and 966 mm, respectively. The soil at Ukulinga farm is loam, fertile and friable, with good drainage and a pH of 4.5. However, it is susceptible to cracking and crusting under flooding. The Bethlehem location is situated at the Agricultural Research Council–Small Grain (latitude: 28°09' S, longitude: 28°18' E). The mean annual temperature and rainfall for Bethlehem was 14.4 ℃ and 702 mm, respectively. The weather data of the locations during the study periods are presented in Table 9.

**Table 9:** Monthly weather data during the field trials at Silverton, Ukulinga and Bethlehem, South Africa, during 2022/2023 growing seasons.

Location	Month	Year	Rainfall (mm)	Tmax (°C)	Tmin (°C)	RH (%)
Silverton	February	2022	34	29	17	62
	March	2022	19	27	16	62
	April	2022	174	25	12	61
	May	2022	16	23	8	56
	June	2022	16	20	5	54
	July	2022	0.76	20	4	53
	August	2022	2.2	23	7	46
Ukulinga	November	2022	121	23	14	79
	December	2022	137	25	15	81
	January	2023	140	25	16	83
	February	2023	118	26	17	82
	March	2023	106	25	16	80
	April	2023	62	23	13	77
	May	2023	32	22	9	70
Bethlehem	November	2022	109	25	11	56
	December	2022	150	25	13	63
	January	2023	148	25	14	68
	February	2023	102	25	14	67
	March	2023	97	24	12	65
	April	2023	55	21	9	62
	May	2023	27	18	5	56

Tmax = average maximum temperature, Tmin = average minimum temperature, RH = relative humidity.

### Experimental design and field trial establishment

The 50 sorghum genotypes were field evaluated using a 5 × 10 alpha lattice design with two replications. Each genotype was planted on a two-meter-long row with inter-row spacing of 90 cm and intra-row spacing of 25 cm. Two seeds were planted and later thinned to one plant. Standard agronomic practices were kept constant in all three sites according to sorghum production guidelines in South Africa^67^. Supplementary irrigation was used to maintain optimum soil moisture conditions throughout the cropping season.

### Data collection

#### Agronomic traits

Data were collected on the following agronomic parameters: days to 50% heading recorded as the number of days from planting to when 50% of the genotypes in each plot had fully exerted panicles; days to 50% maturity recorded as the number of days from planting to when 50% of the genotypes in each plot had dried panicles; biomass production (root and shoot); and grain yield. Shoot biomass (SB) was recorded as the total mass of the above-ground biomass cut from the base of the plant, excluding the grain. The shoots were oven-dried at 70 °C for 48 h, weighed and expressed in g plant^-1^. Root biomass (RB) was recorded as the total root dry matter harvested per genotype per plot. Root samples for each plot were harvested to a depth of 50 cm. The roots were separated from the soil by hand and washed under running water to remove all soil particles. The remaining soil was mixed with water and the suspension was sieved through a 2 mm sieve. Fine roots were collected from the sieve residue and added to the large roots. The roots were oven-dried at 60 °C for 72 h. The dried roots were weighed on a balance to get the RB which was adjusted to g plant^-1^. Total plant biomass (PB) was the sum of all dry plant material for each genotype including RB and SB harvested from the test plots and recorded in g plant^-1^. Root to shoot biomass ratio (RS) was the ratio of the root to shoot biomass as recorded above. Grain yield (GY) was the weight of harvested grain at 12.5% moisture content per genotype per plot and expressed in g plant^-1^. Harvest index (HI) was also calculated using the following formula and expressed in percent:

1 $$HI=\frac{GY}{SB+GY} x 100$$

where HI is the harvest index (%), GY the grain yield (g plant^-1^), and SB is the shoot biomass (g plant^-1^).

#### Carbon stocks determination

Due to the high cost of carbon analysis, the 50 genotypes were sub-sampled, and a select number were retained. Twenty-five genotypes were selected from the Silverton trials based on their grain yield performance and subjected to carbon analysis using two replications. Among the 25 selections, 10 were the top, and 10 were the bottom performing, while five genotypes were random samples.

The carbon analysis involved collecting shoot samples to determine shoot carbon content (SCc), root samples for root carbon content (RCc), and grain samples for grain carbon content (GCc). These samples were oven-dried at 70 °C for 48 h and transformed into fine powder, weighing five grams each. The shoots were pulverized into fine powder using a blender, while the roots and grains were processed into fine powder using a ZM 200 ultra centrifugal mill. The total carbon content of shoot, root, and grain samples was determined by combustion using a LECO TruMac CNS Analyzer^68^.

The shoot (SCs), root (RCs), and grain (GCs) carbon stocks were defined as the total amount of C measured in the respective plant parts according to^69^. These C stocks in the two parts (SCs and RCs) were summed up to derive total plant carbon stocks (PCs). The carbon stocks were calculated based on the carbon content and corresponding biomasses using the following formulas:

2 $$SCs=\frac{SCc}{100}*SB$$

3 $$RCs=\frac{RCc}{100}*RB$$

4 $$GCs =\frac{GCc}{100}*GY$$

where SCs is the shoot carbon stock (g plant^-1^), RCs is the root carbon stock (g plant^-1^), GCs is the grain carbon stock (g plant^-1^), SCc is the shoot carbon content (%), RCc is the root carbon content (%), GCc is the grain carbon content (%), SB is the shoot biomass (g plant^-1^), RB is the root biomass (g plant^-1^), and GY is the grain yield (g plant^-1^).

### Data analysis

The data collected from the 50 genotypes for the agronomic traits and 25 selected genotypes for carbon storage traits were analyzed separately. A combined analysis of variance was performed after homogeneity of variance test procedure^70^ using the Statistical Analysis System (SAS) software version 9.4 program using the PROC general linear model (GLM) procedure^71^ (http://support.sas.com/learn/). The mean values of the test genotypes for the measured traits were compared using Fisher's least significant difference (LSD) procedure at the 5% significance level. Data were subjected to parametric and non-parametric analyses using IBM SPSS statistics 29.0 program^72^ (https://doi.org/10.4324/9781003117452). The rotated component matrix and principal component analysis (PCA) biplots were generated for agronomic and carbon storage traits using the R software version 4.2.3^73^ (https://www.R-project.org/). A hierarchical cluster analysis based on the agglomerative clustering method was also performed using R software to establish genetic relationships among genotypes.

### Ethical approval

Experimental research and field studies on plants, including the collection of plant material, complies with relevant institutional, national, and international guidelines and legislation.

### Supplementary Information

Supplementary Table S1.

## Data Availability

The datasets generated and analyzed during the current study are available from the corresponding author on reasonable request.

## References

[1] Khoddami A, Messina V, VadabalijaVenkata K, Farahnaky A, Blanchard CL, Roberts TH (2023). Sorghum in foods: Functionality and potential in innovative products. Crit. Rev. Food Sci. Nutr..

[2] Tonapi, V. A., Talwar, H. S., Are, A. K., Bhat, B. V., Reddy, C. R. & Dalton, T. J. Sorghum in the 21st Century: Food, Fodder, Feed, Fuel for a Rapidly Changing World. Springer (2020).

[3] Muimba-Kankolongo, A. Food crop production by smallholder farmers in Southern Africa: Challenges and opportunities for improvement (2018).

[4] Xiang Y, Deng Q, Duan H, Guo Y (2017). Effects of biochar application on root traits: a meta-analysis. GCB Bioenergy.

[5] Ritchie, H. & Roser, M. Forests and deforestation. Our world in data (2021).

[6] Janzen HH (2015). Beyond carbon sequestration: soil as conduit of solar energy. Eur. J. Soil Sci..

[7] Paustian K, Lehmann J, Ogle S, Reay D, Robertson GP, Smith P (2016). Climate-smart soils. Nature.

[8] Poeplau C, Don A (2015). Carbon sequestration in agricultural soils via cultivation of cover crops—A meta-analysis. Agric. Ecosyst. Environ..

[9] Brüggemann N, Gessler A, Kayler Z, Keel SG, Badeck F, Barthel M, Boeckx P, Buchmann N, Brugnoli E, Esperschütz J (2011). Carbon allocation and carbon isotope fluxes in the plant-soil-atmosphere continuum: A review. Biogeosciences.

[10] Kätterer T, Bolinder MA, Andrén O, Kirchmann H, Menichetti L (2011). Roots contribute more to refractory soil organic matter than above-ground crop residues, as revealed by a long-term field experiment. Agric. Ecosyst. Environ..

[11] Rasse DP, Rumpel C, Dignac MF (2005). Is soil carbon mostly root carbon? Mechanisms for a specific stabilisation. Plant Soil.

[12] Ghafoor A, Poeplau C, Kätterer T (2017). Fate of straw-and root-derived carbon in a Swedish agricultural soil. Biol. Fertil. Soils.

[13] Menichetti L, Ekblad A, Kätterer T (2015). Contribution of roots and amendments to soil carbon accumulation within the soil profile in a long-term field experiment in Sweden. Agric. Ecosyst. Environ..

[14] Zhang W, Liu K, Wang J, Shao X, Xu M, Li J, Wang X, Murphy DV (2015). Relative contribution of maize and external manure amendment to soil carbon sequestration in a long-term intensive maize cropping system. Sci. Rep..

[15] Rumpel, C., Chabbi, A. & Marschner, B. Carbon storage and sequestration in subsoil horizons: knowledge, gaps, and potentials. *Recarbonization of the biosphere: ecosystems and the global carbon cycle* 445–464 (2012).

[16] Sanaullah M, Chabbi A, Maron PA, Baumann K, Tardy V, Blagodatskaya E, Kuzyakov Y, Rumpel C (2016). How do microbial communities in top-and subsoil respond to root litter addition under field conditions?. Soil Biol. Biochem..

[17] Fan J, McConkey BG, Liang BC, Angers DA, Janzen HH, Kröbel R, Cerkowniak DD, Smith WN (2019). Increasing crop yields and root input make Canadian farmland a large carbon sink. Geoderma.

[18] Russell AE, Cambardella CA, Laird DA, Jaynes DB, Meek DW (2009). Nitrogen fertilizer effects on soil carbon balances in Midwestern US agricultural systems. Ecol. Appl..

[19] Lynch JP, Wojciechowski T (2015). Opportunities, and challenges in the subsoil: Pathways to deeper rooted crops. J. Exp. Bot..

[20] Pierret A, Maeght J-L, Clément C, Montoroi JP, Hartmann C, Gonkhamdee S (2016). Understanding deep roots and their functions in ecosystems: An advocacy for more unconventional research. Ann. Bot..

[21] Mathew I, Shimelis H, Mutema M, Minasny B, Chaplot V (2020). Crops for increasing soil organic carbon stocks—A global meta-analysis. Geoderma.

[22] Bolinder MA, Angers DA, Dubuc JP (1997). Estimating shoot to root ratios and annual carbon inputs in soils for cereal crops. Agric. Ecosyst. Environ..

[23] Manna MC, Swarup A, Wanjari RH, Ravankar HN, Mishra B, Saha MN, Singh YV, Sahi DK, Sarap PA (2005). Long-term effect of fertilizer and manure application on soil organic carbon storage, soil quality and yield sustainability under sub-humid and semi-arid tropical India. Field Crops Res..

[24] Chaplot V, Mathew I, Clulow A, Shimelis H (2023). Are there wheat cultivars allowing enhanced carbon allocation to soils?. Appl. Biosci..

[25] Ahmed IAM, Ortas I, Yucel C, Oktem A, Yucel D, Iqbal MT (2020). Root traits and carbon input by sweet sorghum genotypes differs in two climatic conditions. Aust. J. Crop Sci..

[26] Liang XG, Gao Z, Shen S, Paul MJ, Zhang L, Zhao X, Lin S, Wu G, Chen XM, Zhou SL (2020). Differential ear growth of two maize varieties to shading in the field environment: Effects on whole plant carbon allocation and sugar starvation response. J. Plant Physiol..

[27] Amujoyegbe, B.J., Opabode, J.T., & Olayinka, A., Effect of organic and inorganic fertilizer on yield and chlorophyll content of maize (*Zea mays* L.) and sorghum *Sorghum bicolor* (L.) Moench. *Afr. J. Biotechnol.* 6 (2007).

[28] Mangena, P., Shimelis, H., & Laing, M. Characterisation of sweet stem sorghum genotypes for bio-ethanol production. *Acta Agric. Scand. B Soil Plant Sci.* **68,** 323–333 (2018).

[29] Abraha, T., Githiri, S. M., Kasili, R., Araia, W. & Nyende, A. B. Genetic variation among sorghum (*Sorghum bicolor* L. Moench) landraces from Eritrea under post-flowering drought stress conditions. *Am. J. Plant Sci,* **6,** 1410 (2015).

[30] Shamuyarira KW, Shimelis H, Figlan S, Chaplot V (2022). Path coefficient and principal component analyses for biomass allocation, drought tolerance and carbon sequestration potential in wheat. Plants.

[31] Mulima, E., Sibiya, J., Musvosvi, C. & Nhamucho, E. Identification of important morphological traits in Mozambican sorghum [*Sorghum bicolor* (L.) Moench] germplasm using multivariate analysis. *Afr. J. Agric. Res.* **13,** 1796–1810 (2018).

[32] Enyew, M., Feyissa, T., Geleta, M., Tesfaye, K., Hammenhag, C. & Carlsson, A. S. Genotype by environment interaction, correlation, AMMI, GGE biplot and cluster analysis for grain yield and other agronomic traits in sorghum (*Sorghum bicolor* L. Moench). *PLoS ONE* **16,** e0258211 (2021). 10.1371/journal.pone.0258211 PMC8491923 34610051

[33] Mutava RN, Prasad PVV, Tuinstra MR, Kofoid KD, Yu J (2011). Characterization of sorghum genotypes for traits related to drought tolerance. Field Crops Res..

[34] Gratani, L. Plant phenotypic plasticity in response to environmental factors. *Adv. Bot.* 2014 (2014).

[35] Datta A, Mandal B, Badole S, Majumder SP, Padhan D, Basak N, Barman A, Kundu R, Narkhede WN (2018). Interrelationship of biomass yield, carbon input, aggregation, carbon pools and its sequestration in Vertisols under long-term sorghum-wheat cropping system in semi-arid tropics. Soil Tillage Res..

[36] Boyles RE, Brenton ZW, Kresovich S (2019). Genetic and genomic resources of sorghum to connect genotype with phenotype in contrasting environments. Plant J..

[37] George-Jaeggli B, Jordan DR, van Oosterom EJ, Hammer GL (2011). Decrease in sorghum grain yield due to the dw3 dwarfing gene is caused by reduction in shoot biomass. Field Crops Res..

[38] Reynolds MP, Pellegrineschi A, Skovmand B (2005). Sink-limitation to yield and biomass: a summary of some investigations in spring wheat. Ann. Appl. Boil..

[39] Haussmann, B. I. G., Fred Rattunde, H., Weltzien‐Rattunde, E., Traoré, P. S. C., Vom Brocke, K., & Parzies, H. K. Breeding strategies for adaptation of pearl millet and sorghum to climate variability and change in West Africa. *J. Agron. Crop Sci*. **198,** 327–339 (2012).

[40] Abreha KB, Enyew M, Carlsson AS, Vetukuri RR, Feyissa T, Motlhaodi T, Geleta M (2022). Sorghum in dryland: Morphological, physiological, and molecular responses of sorghum under drought stress. Planta.

[41] Fracasso A, Trindade LM, Amaducci S (2016). Drought stress tolerance strategies revealed by RNA-Seq in two sorghum genotypes with contrasting WUE. BMC Plant Biol..

[42] Sukumaran S, Li X, Li X, Zhu C, Bai G, Perumal R, Yu J (2016). QTL mapping for grain yield, flowering time, and stay-green traits in sorghum with genotyping-by-sequencing markers. Crop Sci..

[43] Getnet, Z. G., Azamal Husen, A. H., Masresha Fetene, M. F., & Gietahun Yemata, G. Y. Growth, water status, physiological, biochemical and yield response of Stay Green sorghum (*Sorghum bicolor* [L.] Moench) varieties-a field trial under drought-prone area in Amhara Regional State, Ethiopia. *J. Agron.* **14,** 188–203 (2015).

[44] Figueroa-Bustos V, Palta JA, Chen Y, Siddique KHM (2019). Early season drought largely reduces grain yield in wheat cultivars with smaller root systems. Plants.

[45] Chai Q, Gan Y, Zhao C, Xu H-L, Waskom RM, Niu Y, Siddique KHM (2016). Regulated deficit irrigation for crop production under drought stress. A review. Agron. Sustain. Dev..

[46] Fang Y, Du Y, Wang J, Wu A, Qiao S, Xu B, Zhang S, Siddique KHM, Chen Y (2017). Moderate drought stress affected root growth and grain yield in old, modern, and newly released cultivars of winter wheat. Front. Plant Sci..

[47] Cunniff, J., Purdy, S. J., Barraclough, T. J. P., Castle, M., Maddison, A. L., Jones, L. E., Shield, I. F., Gregory, A. S., & Karp, A. High yielding biomass genotypes of willow (*Salix spp.)* show differences in below ground biomass allocation. *Biomass Bioenergy* **80,** 114–127 (2015) 10.1016/j.biombioe.2015.04.020 PMC4547486 26339128

[48] Kusalkar DV, Awari VR, Pawar VY, Shinde MS (2003). Physiological parameters in relation to grain yield in rabi sorghum on medium soil. Adv. Plant Sci..

[49] Borrás L, Slafer GA, Otegui ME (2004). Seed dry weight response to source sink manipulations in wheat, maize, and soybean: A quantitative reappraisal. Field Crops Res..

[50] Singh, M., Guleria, N., Prakasa Rao, E. V. S. & Goswami, P. Efficient C sequestration and benefits of medicinal vetiver cropping in tropical regions. *Agron. Sustain Dev.* **34,** 603–607 (2014).

[51] Monti A, Zatta A (2009). Root distribution and soil moisture retrieval in perennial and annual energy crops in Northern Italy. Agric. Ecosyst. Environ..

[52] Bonifas KD, Lindquist JL (2009). Effects of nitrogen supply on the root morphology of corn and velvetleaf. J. Plant Nutr..

[53] Andreas, P., Kisiala, A., Emery, R. J. N., De Clerck-Floate, R., Tooker, J. F., Price, P. W., Miller Iii, D. G., Chen, M. S. & Connor, E. F. Cytokinins are abundant and widespread among insect species. *Plants* **9**, 208 (2020). 10.3390/plants9020208 PMC7076654 32041320

[54] Qi Y, Wei W, Chen C, Chen L (2019). Plant root-shoot biomass allocation over diverse biomes: A global synthesis. Glob. Ecol. Conserv..

[55] Brassard BW, Chen HYH, Bergeron Y (2009). Influence of environmental variability on root dynamics in northern forests. Crit. Rev. Plant Sci..

[56] Klimešová J, Martínková J, Ottaviani G (2018). Belowground plant functional ecology: towards an integrated perspective. Funct. Ecol..

[57] Prasad PVV, Staggenborg SA, Ristic Z (2008). Impacts of drought and/or heat stress on physiological, developmental, growth, and yield processes of crop plants Response of crops to limited water. Underst. Model. Water Stress Eff. Plant Growth Process..

[58] Larson JE, Anacker BL, Wanous S, Funk JL (2020). Ecological strategies begin at germination: Traits, plasticity, and survival in the first four days of plant life. Funct. Ecol..

[59] Tolbert VR, Todd DE, Mann LK, Jawdy CM, Mays DA, Malik R, Bandaranayake W, Houston A, Tyler D, Pettry DE (2002). Changes in soil quality and below-ground carbon storage with conversion of traditional agricultural crop lands to bioenergy crop production. Environ. Pollut..

[60] Ayana, A. & Bekele, E. Multivariate analysis of morphological variation in sorghum (*Sorghum bicolor* (L.) Moench) germplasm from Ethiopia and Eritrea. *Genet. Resour. Crop Evol.* **46,** 273–2841 (999).

[61] Upadhyaya HD, Reddy KN, Gowda CLL, Singh S (2007). Phenotypic diversity in the pigeonpea (*Cajanus*
*cajan*) core collection. Genet. Resour. Crop Evol..

[62] Saroj SK, Singh MN, Kumar R, Singh T, Singh MK (2013). Genetic variability, correlation, and path analysis for yield attributes in pigeon pea. BioScan.

[63] Seetharam K, Ganesamurthy K (2013). Characterization of sorghum genotypes for yield and other agronomic traits through genetic variability and diversity analysis. Electron. J. Plant Breed..

[64] Billot C, Ramu P, Bouchet S, Chantereau J, Deu M, Gardes L, Noyer JL, Rami JF, Rivallan R, Li Y (2013). Massive sorghum collection genotyped with SSR markers to enhance use of global genetic resources. PLoS One.

[65] Sorkheh K, Shiran B, Gradziel TM, Epperson BK, Martínez-Gómez P, Asadi E (2007). Amplified fragment length polymorphism as a tool for molecular characterization of almond germplasm: Genetic diversity among cultivated genotypes and related wild species of almond, and its relationships with agronomic traits. Euphytica.

[66] Nevo E, Chen G (2010). Drought and salt tolerances in wild relatives for wheat and barley improvement. Plant Cell Environ..

[67] Department of Agriculture, Forestry and Fisheries (DAFF). Sorghum production guidelines. Pretoria (2010).

[68] Rayment, G.E., & Lyons, D.J. Soil chemical methods: Australasia. *CSIRO Publishing* **3** (2011)

[69] Grewer U, Nash J, Gurwick N, Bockel L, Galford G, Richards M, Junior CC, White J, Pirolli G, Wollenberg E (2018). Analyzing the greenhouse gas impact potential of smallholder development actions across a global food security program. Environ. Res. Lett..

[70] Levene, H. Robust tests for equality of variances. Contributions to probability and statistics, 278–292 (1960).

[71] Guide, P. SAS® 9.4 Output Delivery System. (2014).

[72] Pallant, J. SPSS survival manual: A step by step guide to data analysis using IBM SPSS. *Routledge*, (2020).

[73] Team, R. Core. "R: A language and environment for statistical computing. R Foundation for Statistical Computing."(2013).

