# Supplementary material for: Response of Sorghum bicolor genotypes for yield and yield components and organic carbon storage in the shoot and root systems
Source: Sci Rep. 2024 Apr 25;14:9499. doi: 10.1038/s41598-024-59956-x (PMC11045799; doi:10.1038/s41598-024-59956-x)
Supplement: Supplementary file 1 — Supplementary Table S1. [file 41598_2024_59956_MOESM1_ESM.docx]

| Genotype | DTH | | | DTM | | | PH | | | PB | | | SB | | | RB | | | RS | | | GY | | | HI | | |
| --- | --- | --- | --- | --- | --- | --- | --- | --- | --- | --- | --- | --- | --- | --- | --- | --- | --- | --- | --- | --- | --- | --- | --- | --- | --- | --- | --- |
|  | **STN** | **UKN** | **BTH** | **STN** | **UKN** | **BTH** | **STN** | **UKN** | **BTH** | **STN** | **UKN** | **BTH** | **STN** | **UKN** | **BTH** | **STN** | **UKN** | **BTH** | **STN** | **UKN** | **BTH** | **STN** | **UKN** | **BTH** | **STN** | **UKN** | **BTH** |
| 05-POTCH-138 | 77 | 68 | 76 | 154 | 139 | 139 | 107.67 | 112.83 | 145.33 | 27.14 | 27.56 | 24.07 | 9.79 | 7.78 | 9.14 | 17.35 | 19.78 | 14.93 | 1.85 | 2.54 | 1.71 | 6.50 | 8.17 | 7.33 | 40.23 | 51.07 | 44.81 |
| 16MZ | 91 | 65 | 61 | 142 | 140 | 142 | 155.50 | 168.67 | 161.33 | 34.21 | 34.47 | 33.71 | 22.75 | 23.02 | 22.24 | 11.46 | 11.45 | 11.46 | 0.51 | 0.50 | 0.51 | 12.11 | 12.11 | 12.11 | 35.05 | 34.48 | 35.26 |
| AS106 | 87 | 83 | 73 | 139 | 136 | 136 | 170.00 | 166.50 | 174.83 | 30.14 | 28.80 | 29.39 | 8.52 | 8.37 | 8.37 | 21.62 | 20.43 | 21.02 | 2.54 | 2.44 | 2.55 | 7.55 | 8.44 | 9.34 | 46.81 | 50.21 | 52.76 |
| AS108 | 79 | 85 | 69 | 134 | 139 | 148 | 136.00 | 143.17 | 148.50 | 32.79 | 25.63 | 30.68 | 12.99 | 10.66 | 13.29 | 19.80 | 14.98 | 17.39 | 1.52 | 1.38 | 1.32 | 8.92 | 10.00 | 7.84 | 40.75 | 48.73 | 36.93 |
| AS109 | 103 | 82 | 107 | 130 | 132 | 133 | 237.50 | 142.33 | 211.83 | 34.50 | 32.72 | 33.61 | 19.50 | 18.97 | 19.23 | 15.00 | 13.75 | 14.38 | 0.88 | 0.73 | 0.75 | 10.67 | 15.66 | 13.16 | 36.57 | 45.23 | 40.63 |
| AS111 | 83 | 97 | 73 | 162 | 134 | 136 | 151.00 | 244.17 | 161.83 | 28.89 | 29.71 | 11.07 | 16.86 | 17.58 | 7.48 | 12.03 | 12.13 | 3.59 | 0.71 | 0.71 | 0.49 | 2.39 | 2.68 | 2.53 | 12.41 | 13.38 | 25.27 |
| AS113 | 91 | 73 | 81 | 135 | 146 | 131 | 205.00 | 125.50 | 232.17 | 28.60 | 34.52 | 25.30 | 16.69 | 24.00 | 12.50 | 11.91 | 10.52 | 12.81 | 0.71 | 0.44 | 1.63 | 10.56 | 15.09 | 11.71 | 38.75 | 38.54 | 53.09 |
| AS114 | 91 | 65 | 66 | 151 | 131 | 133 | 126.33 | 140.00 | 136.67 | 21.85 | 30.34 | 24.87 | 17.65 | 22.04 | 17.54 | 4.20 | 8.31 | 7.34 | 0.24 | 0.39 | 0.42 | 13.46 | 15.07 | 11.85 | 43.36 | 40.88 | 40.19 |
| AS115 | **89** | **81** | **95** | **141** | **125** | **123** | **126.83** | **145.67** | **275.00** | **16.90** | **18.07** | **29.22** | **7.48** | **12.92** | **17.22** | **9.42** | **5.14** | **12.00** | **1.26** | **0.41** | **0.70** | **29.66** | **15.80** | **29.78** | **79.86** | **55.15** | **63.36** |
| AS116 | 69 | 94 | 69 | 134 | 149 | 131 | 152.83 | 150.83 | 122.67 | 17.86 | 24.22 | 22.49 | 14.45 | 15.45 | 14.78 | 3.41 | 8.78 | 7.71 | 0.24 | 0.57 | 0.53 | 4.11 | 5.22 | 3.73 | 22.16 | 24.99 | 20.56 |
| AS117 | 65 | 68 | 77 | 140 | 141 | 136 | 132.17 | 92.17 | 197.50 | 29.07 | 24.74 | 30.44 | 20.99 | 17.33 | 22.97 | 8.08 | 7.41 | 7.48 | 0.39 | 0.43 | 0.32 | 11.28 | 12.32 | 10.23 | 34.96 | 41.40 | 31.00 |
| AS121 | 72 | 94 | 87 | 134 | 135 | 132 | 164.33 | 156.33 | 172.33 | 28.95 | 26.03 | 23.12 | 18.72 | 18.72 | 18.72 | 10.23 | 7.31 | 4.40 | 0.55 | 0.41 | 0.24 | 6.81 | 7.43 | 10.23 | 24.34 | 28.93 | 35.34 |
| AS122 | 69 | 65 | 73 | 142 | 142 | 133 | 128.00 | 147.67 | 143.33 | 42.65 | 44.84 | 43.75 | 23.90 | 23.90 | 23.90 | 18.76 | 20.94 | 19.85 | 0.79 | 0.88 | 0.83 | 8.50 | 8.50 | 8.50 | 26.51 | 26.23 | 26.22 |
| AS129 | 83 | 83 | 88 | 142 | 148 | 140 | 122.67 | 114.33 | 159.83 | 35.43 | 13.98 | 31.27 | 22.89 | 7.48 | 21.86 | 12.54 | 6.50 | 9.42 | 0.55 | 0.87 | 0.43 | 3.78 | 2.89 | 3.18 | 14.17 | 27.87 | 12.69 |
| AS130 | **81** | **65** | **85** | **138** | **148** | **148** | **163.83** | **128.17** | **198.00** | **20.56** | **24.78** | **31.36** | **9.90** | **9.90** | **14.08** | **10.66** | **14.88** | **17.28** | **1.08** | **1.50** | **1.23** | **21.33** | **21.33** | **12.83** | **68.30** | **67.58** | **47.69** |
| AS131 | 89 | 69 | 86 | 132 | 143 | 134 | 170.33 | 110.00 | 199.17 | 30.65 | 28.18 | 28.71 | 20.13 | 20.13 | 20.13 | 10.53 | 8.05 | 8.58 | 0.52 | 0.40 | 0.43 | 7.67 | 7.00 | 8.34 | 27.95 | 25.40 | 29.24 |
| AS132 | **106** | **98** | **80** | **136** | **136** | **126** | **238.17** | **103.83** | **171.17** | **32.54** | **26.48** | **18.69** | **20.69** | **14.45** | **11.69** | **11.85** | **12.03** | **7.00** | **0.61** | **0.89** | **0.60** | **21.57** | **21.36** | **7.75** | **51.74** | **60.09** | **39.87** |
| AS133 | 69 | 82 | 81 | 162 | 138 | 136 | 89.80 | 159.33 | 154.17 | 14.92 | 27.64 | 17.28 | 11.43 | 15.73 | 9.93 | 3.49 | 11.91 | 7.34 | 0.31 | 0.75 | 0.76 | 12.88 | 15.07 | 14.33 | 52.97 | 48.93 | 59.28 |
| AS134 | **83** | **85** | **65** | **135** | **135** | **136** | **195.33** | **132.83** | **160.00** | **39.17** | **42.23** | **25.17** | **21.04** | **20.58** | **19.48** | **18.13** | **21.65** | **5.70** | **0.86** | **1.05** | **0.29** | **26.31** | **27.71** | **10.23** | **55.57** | **57.35** | **34.50** |
| AS135 | 65 | 79 | 69 | 170 | 143 | 141 | 144.67 | 96.75 | 165.17 | 23.71 | 23.24 | 21.49 | 16.63 | 13.84 | 12.29 | 7.08 | 9.40 | 9.20 | 0.44 | 0.68 | 0.75 | 5.20 | 5.20 | 5.20 | 24.11 | 27.32 | 29.76 |
| AS136 | **90** | **87** | **98** | **138** | **162** | **149** | **126.67** | **147.50** | **135.83** | **21.55** | **27.07** | **29.95** | **11.25** | **16.93** | **20.21** | **10.30** | **10.14** | **9.75** | **0.92** | **0.60** | **0.48** | **15.07** | **16.06** | **9.35** | **57.26** | **48.51** | **31.67** |
| AS137 | 88 | 76 | 66 | 141 | 136 | 168 | 123.92 | 129.33 | 141.17 | 23.13 | 31.06 | 19.98 | 15.42 | 20.82 | 13.31 | 7.71 | 10.24 | 6.67 | 0.50 | 0.50 | 0.49 | 14.12 | 8.57 | 12.18 | 47.80 | 29.49 | 47.76 |
| AS138 | **65** | **91** | **77** | **139** | **149** | **134** | **156.67** | **141.67** | **140.17** | **19.72** | **22.01** | **43.90** | **11.93** | **12.97** | **20.19** | **7.79** | **9.04** | **23.71** | **0.65** | **0.70** | **1.20** | **18.54** | **18.89** | **14.92** | **60.84** | **59.36** | **43.16** |
| AS140 | 65 | 96 | 81 | 130 | 134 | 138 | 151.33 | 128.50 | 189.50 | 24.11 | 25.31 | 26.50 | 19.24 | 19.04 | 18.83 | 4.88 | 6.27 | 7.67 | 0.24 | 0.33 | 0.43 | 4.70 | 4.70 | 4.70 | 20.10 | 19.80 | 20.60 |
| AS141 | 63 | 79 | 66 | 132 | 147 | 131 | 174.08 | 102.67 | 142.50 | 23.06 | 25.86 | 25.15 | 18.77 | 18.77 | 18.77 | 4.29 | 7.09 | 6.38 | 0.23 | 0.38 | 0.35 | 13.91 | 13.91 | 13.91 | 42.56 | 42.17 | 44.45 |
| AS143 | 71 | 78 | 81 | 136 | 138 | 133 | 219.67 | 121.17 | 209.17 | 18.41 | 18.53 | 15.00 | 12.10 | 10.13 | 10.36 | 6.31 | 8.40 | 4.64 | 0.53 | 0.85 | 0.45 | 8.75 | 9.16 | 8.34 | 42.04 | 47.47 | 44.63 |
| AS145 | **69** | **100** | **89** | **140** | **147** | **136** | **155.33** | **159.33** | **158.33** | **27.09** | **36.03** | **33.49** | **21.92** | **20.38** | **23.56** | **5.17** | **15.66** | **9.93** | **0.24** | **0.78** | **0.42** | **15.31** | **29.93** | **13.04** | **40.90** | **59.50** | **35.60** |
| AS147 | 88 | 77 | 76 | 132 | 146 | 137 | 165.50 | 116.67 | 153.50 | 15.82 | 14.86 | 17.91 | 7.51 | 6.17 | 8.84 | 8.31 | 8.69 | 9.06 | 1.11 | 1.48 | 1.06 | 4.69 | 4.57 | 4.63 | 38.44 | 43.18 | 34.93 |
| AS148 | 99 | 73 | 85 | 140 | 142 | 132 | 174.00 | 151.83 | 187.00 | 30.38 | 28.46 | 22.67 | 16.23 | 13.47 | 9.90 | 14.15 | 14.99 | 12.77 | 0.88 | 1.11 | 1.29 | 12.58 | 13.08 | 21.33 | 44.05 | 48.58 | 68.31 |
| AS152 | 81 | 66 | 89 | 129 | 129 | 133 | 120.17 | 115.17 | 147.33 | 26.39 | 24.60 | 26.86 | 6.49 | 5.58 | 7.40 | 19.90 | 19.02 | 19.46 | 3.07 | 3.46 | 2.63 | 9.30 | 8.36 | 10.23 | 58.90 | 59.98 | 58.03 |
| AS194 | 66 | 80 | 90 | 148 | 141 | 139 | 136.83 | 90.67 | 173.17 | 37.20 | 41.27 | 17.44 | 25.38 | 27.48 | 10.90 | 11.82 | 13.79 | 6.55 | 0.47 | 0.50 | 0.64 | 13.30 | 15.48 | 18.89 | 34.32 | 35.47 | 63.47 |
| AS203 | **94** | **88** | **65** | **138** | **141** | **114** | **177.67** | **185.17** | **160.67** | **35.28** | **42.65** | **36.65** | **20.54** | **27.91** | **21.91** | **14.74** | **14.74** | **14.74** | **0.73** | **0.52** | **0.68** | **16.63** | **16.63** | **16.63** | **45.02** | **37.72** | **43.20** |
| AS205 | 93 | 105 | 69 | 134 | 136 | 133 | 261.50 | 194.00 | 214.33 | 31.91 | 34.42 | 33.22 | 20.01 | 20.00 | 20.06 | 11.91 | 14.42 | 13.16 | 0.60 | 0.72 | 0.66 | 12.65 | 15.07 | 10.23 | 38.74 | 43.45 | 33.78 |
| AS251 | **86** | **83** | **87** | **148** | **143** | **136** | **144.33** | **120.83** | **129.00** | **26.31** | **27.44** | **29.05** | **14.72** | **14.35** | **14.82** | **11.59** | **13.09** | **14.24** | **0.79** | **0.91** | **0.96** | **21.83** | **21.83** | **21.83** | **59.75** | **59.79** | **59.57** |
| AS391 | 80 | 87 | 86 | 129 | 130 | 132 | 253.33 | 178.00 | 177.17 | 42.18 | 37.54 | 44.22 | 24.14 | 22.41 | 23.27 | 18.04 | 15.13 | 20.95 | 0.75 | 0.74 | 0.90 | 12.22 | 12.22 | 12.14 | 33.63 | 36.02 | 34.26 |
| AS449 | 74 | 73 | 93 | 136 | 145 | 137 | 155.17 | 122.17 | 243.67 | 16.67 | 17.68 | 29.25 | 9.67 | 10.68 | 17.57 | 7.00 | 7.00 | 11.68 | 0.73 | 0.66 | 0.66 | 10.56 | 9.96 | 21.79 | 51.17 | 48.22 | 55.35 |
| AS560 | 69 | 80 | 85 | 137 | 139 | 136 | 178.33 | 126.00 | 140.67 | 33.01 | 30.57 | 35.73 | 20.84 | 18.64 | 23.32 | 12.17 | 11.93 | 12.41 | 0.58 | 0.63 | 0.54 | 11.68 | 14.30 | 12.99 | 35.81 | 43.42 | 35.93 |
| AS563 | **86** | **84** | **82** | **136** | **138** | **132** | **179.67** | **124.67** | **217.50** | **34.68** | **26.13** | **38.25** | **23.11** | **20.70** | **20.12** | **11.57** | **5.43** | **18.13** | **0.50** | **0.26** | **0.90** | **13.28** | **12.31** | **24.91** | **36.48** | **37.29** | **55.27** |
| AS72 | 66 | 75 | 66 | 131 | 142 | 127 | 169.50 | 177.83 | 128.00 | 37.99 | 37.90 | 36.89 | 19.53 | 15.92 | 17.72 | 18.46 | 21.98 | 19.17 | 0.95 | 1.37 | 1.08 | 13.59 | 12.92 | 14.26 | 41.16 | 44.94 | 44.57 |
| AS74 | 77 | 69 | 83 | 136 | 132 | 138 | 149.67 | 139.50 | 152.50 | 41.99 | 38.37 | 33.12 | 15.79 | 13.48 | 23.27 | 26.20 | 24.89 | 9.85 | 1.66 | 1.89 | 0.44 | 14.83 | 14.75 | 14.39 | 48.43 | 52.60 | 38.33 |
| G50 | 65 | 69 | 69 | 122 | 132 | 132 | 147.83 | 165.83 | 175.33 | 19.16 | 22.23 | 19.16 | 7.56 | 7.56 | 7.56 | 11.60 | 14.67 | 11.60 | 1.53 | 1.94 | 1.63 | 9.08 | 8.50 | 9.66 | 54.57 | 52.93 | 56.45 |
| ICS634 | 73 | 74 | 69 | 134 | 139 | 142 | 79.68 | 131.67 | 154.83 | 14.37 | 14.07 | 13.78 | 8.14 | 8.14 | 8.14 | 6.23 | 5.93 | 5.64 | 0.77 | 0.73 | 0.70 | 9.83 | 9.64 | 10.03 | 54.70 | 54.21 | 55.45 |
| ICSV92001 | 77 | 87 | 74 | 142 | 142 | 138 | 150.83 | 124.17 | 187.83 | 33.20 | 23.29 | 19.41 | 23.21 | 12.38 | 10.12 | 9.99 | 10.92 | 9.29 | 0.43 | 0.88 | 0.98 | 7.50 | 15.07 | 15.07 | 24.46 | 55.00 | 60.23 |
| LP4403 | 76 | 76 | 86 | 145 | 135 | 131 | 185.33 | 173.67 | 157.67 | 31.77 | 35.85 | 36.43 | 19.17 | 22.08 | 25.00 | 12.60 | 13.77 | 11.43 | 0.67 | 0.62 | 0.46 | 7.39 | 8.80 | 8.09 | 28.04 | 28.39 | 24.45 |
| MAMOLOKWANE | 84 | 69 | 103 | 144 | 136 | 140 | 191.83 | 99.17 | 203.00 | 20.77 | 26.92 | 19.14 | 12.33 | 16.00 | 10.65 | 8.44 | 10.92 | 8.49 | 0.68 | 0.68 | 0.80 | 12.28 | 15.07 | 10.23 | 49.98 | 48.50 | 49.01 |
| NW5393 | 69 | 80 | 70 | 133 | 135 | 136 | 169.50 | 218.00 | 143.83 | 18.53 | 17.50 | 16.49 | 11.54 | 11.54 | 11.54 | 6.99 | 5.96 | 4.94 | 0.61 | 0.52 | 0.44 | 13.00 | 13.00 | 13.00 | 52.97 | 52.97 | 53.60 |
| NW5430 | 101 | 99 | 86 | 136 | 123 | 141 | 173.17 | 140.50 | 158.08 | 23.54 | 22.49 | 21.44 | 16.08 | 16.08 | 16.08 | 7.45 | 6.41 | 5.36 | 0.47 | 0.40 | 0.33 | 7.35 | 8.79 | 10.23 | 31.39 | 35.34 | 38.88 |
| PAN8816 | 76 | 69 | 81 | 132 | 146 | 142 | 117.83 | 96.33 | 107.00 | 30.30 | 36.19 | 34.27 | 16.61 | 17.64 | 18.15 | 13.69 | 18.55 | 16.12 | 0.82 | 1.05 | 0.90 | 3.75 | 3.75 | 3.75 | 17.89 | 17.56 | 17.28 |
| SS27 | **66** | **106** | **65** | **142** | **151** | **133** | **139.83** | **109.25** | **158.67** | **55.91** | **31.92** | **28.12** | **41.86** | **16.55** | **16.21** | **14.05** | **15.36** | **11.91** | **0.47** | **1.04** | **0.73** | **17.58** | **17.58** | **17.58** | **29.58** | **51.51** | **52.03** |
| SV07002 | 71 | 85 | 77 | 142 | 138 | 136 | 167.50 | 188.83 | 107.50 | 25.54 | 24.72 | 29.82 | 16.38 | 15.83 | 19.38 | 9.17 | 8.89 | 10.44 | 0.57 | 0.57 | 0.54 | 6.50 | 6.31 | 7.03 | 28.38 | 28.50 | 26.55 |
| Mean | 79.14 | 80.60 | 78.81 | 138.92 | 139.25 | 135.86 | 160.29 | 140.02 | 167.72 | 28.21 | 28.22 | 27.38 | 16.81 | 16.05 | 16.08 | 11.40 | 12.17 | 11.30 | 0.79 | 0.90 | 0.81 | 11.66 | 12.33 | 11.71 | 40.52 | 42.71 | 41.71 |
| SD | 11.31 | 10.92 | 10.49 | 8.78 | 6.92 | 7.49 | 37.33 | 32.13 | 33.58 | 8.45 | 7.42 | 7.86 | 6.26 | 5.42 | 5.27 | 5.00 | 4.90 | 4.96 | 0.55 | 0.62 | 0.51 | 5.69 | 5.84 | 5.55 | 14.00 | 12.57 | 13.14 |
| SE | 1.60 | 1.54 | 1.48 | 1.24 | 0.98 | 1.06 | 5.28 | 4.54 | 4.75 | 1.20 | 1.05 | 1.11 | 0.89 | 0.77 | 0.75 | 0.71 | 0.69 | 0.70 | 0.08 | 0.09 | 0.07 | 0.81 | 0.83 | 0.78 | 1.98 | 1.78 | 1.86 |
| Skewness | 0.41 | 0.45 | 0.43 | 1.44 | 0.30 | 1.17 | 0.66 | 0.89 | 0.85 | 0.61 | 0.20 | 0.13 | 0.98 | -0.01 | -0.12 | 0.66 | 0.63 | 0.61 | 2.25 | 2.17 | 1.90 | 0.94 | 0.73 | 0.94 | 0.26 | -0.34 | -0.04 |
| kurtosis | -0.72 | -0.50 | -0.19 | 3.03 | 1.25 | 6.82 | 0.95 | 1.15 | 1.10 | 0.84 | -0.31 | -0.44 | 3.57 | -0.55 | -1.29 | 0.35 | -0.26 | -0.38 | 6.51 | 5.75 | 4.39 | 1.31 | 0.93 | 1.48 | 0.10 | -0.61 | -0.68 |

**Table S1** Mean values for nine agronomic traits of the 50 sorghum genotypes evaluated at Silverton (STN), Ukulinga (UKN) and Bethlehem (BTH) in 2022 and 2023 growing seasons in South Africa. SD = standard deviation, SE = standard error, DTH = days to 50% heading, DTM = days to 50% maturity, PH = plant height (cm), PB = total plant biomass (g plant^-1^), SB = shoot biomass (g plant^-1^), RB = root biomass (g plant^-1^), RS = root to shoot biomass ratio, GY = grain yield (g plant^-1^), HI = harvest index (%). The top ten performing genotypes at each location
